# Hypomethylation associated enhanced transcription of trefoil factor-3 mediates tamoxifen-stimulated oncogenicity of ER+ endometrial carcinoma cells

**DOI:** 10.18632/oncotarget.20461

**Published:** 2017-08-24

**Authors:** Vijay Pandey, Min Zhang, Qing-Yun Chong, Mingliang You, Ainiah Rushdiana Raquib, Amit K. Pandey, Dong-Xu Liu, Liang Liu, Lan Ma, Sudhakar Jha, Zheng-Sheng Wu, Tao Zhu, Peter E. Lobie

**Affiliations:** ^1^ Cancer Science Institute of Singapore, National University of Singapore, Singapore; ^2^ Department of Pharmacology, National University of Singapore, Singapore; ^3^ Hefei National Laboratory for Physical Sciences at Microscale and School of Life Sciences, University of Science and Technology of China, Hefei, P.R. China; ^4^ Department of Pathology, Anhui Medical University, Hefei, P.R China; ^5^ Tsinghua Berkeley Shenzhen Institute, Division of Life Sciences & Health, Tsinghua University Graduate School, Shenzhen, P.R China; ^6^ Department of Oncology, Fudan University Shanghai Cancer Center, Fudan University, Shanghai, P.R China; ^7^ Department of Radiology, Fudan University Shanghai Cancer Center, Fudan University, Shanghai, P.R China; ^8^ School of Science, Auckland University of Technology, Auckland, New Zealand

**Keywords:** TFF3, tamoxifen, endometrial carcinoma, breast cancer, oestrogen receptor (ER)

## Abstract

Tamoxifen (TAM) is widely used as an adjuvant therapy for women with breast cancer (BC). However, TAM possesses partial oestrogenic activity in the uterus and its use has been associated with an increased incidence of endometrial carcinoma (EC). The molecular mechanism for these observations is not well understood. Herein, we demonstrated that forced expression of *Trefoil factor 3* (*TFF3)*, in oestrogen receptor-positive (ER+) EC cells significantly increased cell cycle progression, cell survival, anchorage-independent growth, invasiveness and tumour growth in xenograft models. Clinically, elevated TFF3 protein expression was observed in EC compared with normal endometrial tissue, and its increased expression in EC was significantly associated with myometrial invasion. TAM exposure increased expression of TFF3 in ER+ EC cells and its elevated expression resulted in increased oncogenicity and invasiveness. TAM-stimulated expression of TFF3 in EC cells was associated with hypomethylation of the *TFF3 promoter sequence* and c-JUN/SP1-dependent transcriptional activation. In addition, *small interfering* (*si) RNA*-mediated depletion or polyclonal antibody inhibition of TFF3 significantly abrogated oncogenicity and invasiveness in EC cells consequent to TAM induction or forced expression of TFF3. Hence, TAM-stimulated upregulation of *TFF3* in EC cells was critical in promoting EC progression associated with TAM treatment. Importantly, inhibition of TFF3 function might be an attractive molecular modality to abrogate the stimulatory effects of TAM on endometrial tissue and to limit the progression of EC.

## INTRODUCTION

Endometrial carcinoma (EC) is the most common gynaecological malignancy and exhibits an unacceptable rate of recurrence both locally and distally [1] [2]. The majority of ECs are classified as type I oestrogen-dependent endometrioid adenocarcinomas in which oestrogen has been identified as the predominant aetiological factor, whereas type II ECs are represented largely by serous and clear-cell adenocarcinomas [3]. Surgery represents a favourable option for treatment of EC cases detected at early stages. However, tumours identified at later stages are associated with high levels of morbidity and mortality with 87% recurrence and distal metastases occurring within three years of primary treatment [4]. Based on epidemiological studies, oestrogen signalling has been identified as a critical promoter of endometrial carcinogenesis [5]. It has been observed that oestrogenic signalling is able to contribute to the progression of breast cancer (BC) independent of oestrogen (E2) [6]. Studies have identified various agonists that can initiate the oestrogenic signalling pathway including plant-based phytoestrogens, chemicals such as bisphenol-A (BPA) and therapeutic agents such as selective oestrogen receptor modulators (SERMs) [5, 7, 8].

Tamoxifen (TAM), was the first SERM available for clinical use and has been regarded as a highly efficacious agent for the treatment of BC, treatment of metastatic BC, and reduction in BC incidence in high-risk women [9] [10]. Although TAM acts to inhibit ERα in breast, it was observed to have mild agonist activity in the endometrium, skeletal and cardiovascular systems [11, 12]. These partial oestrogenic actions of TAM produce beneficial effects on the skeletal and the cardiovascular system in postmenopausal women but are also associated with, endometrial hyperplasia, and EC [13].

Trefoil factor 3 (TFF3), an oestrogen responsive gene, is one of three members grouped in the trefoil factor family that normally functions to protect the intestinal mucosal surface and promote epithelial healing in times of injury [14]. Recently, TFF3 has emerged as a clinically validated and functionally potent target in oncology. Whilst there may be tissue-specific effects, an increasing number of studies have shown that TFF3 promotes cell survival and cell cycle progression, angiogenesis and metastatic dissemination in various cancers, acting through signalling mediators such as epidermal growth factor receptor (EGFR), AKT, mitogen-activated protein kinase (MAPK), signal transducer and activator of transcription 3 (STAT3) and the E-CADHERIN-CATENIN complex [15–21]. TFF3 expression is absent or relatively low in normal human female reproductive tissues, with transient increased expression in the proliferative phase of the endometrium [22]. In contrast, TFF3 has been observed to be prominently elevated in cervical [23], endometrial [24] and mammary carcinoma [17, 25]. Elevated levels of TFF3 protein were observed to be associated with advanced clinicopathological features of disease, such as tumour size, higher disease grade and metastases [21, 25]. Moreover, TFF3 has been identified as the gene most highly associated with high grade EC [24]. TFF3 expression is significantly correlated with survival outcome of patients also with ER+ BC treated with TAM [17]. Subsequent studies also found a positive association between TFF3 and its interaction with type I oestrogen-dependent endometrioid adenocarcinoma [26].

Herein, we sought to determine the role of TFF3 protein in promoting oncogenicity and invasiveness of ER+ EC cells. We also assessed the involvement of TFF3 in TAM-stimulated EC progression. Furthermore, by elucidation of the mechanism via which TAM can regulate TFF3 expression, we aim to shed light on the mechanism of TAM-stimulated EC.

## RESULTS

### TFF3 stimulates oncogenicity of EC cells

**Figure 1:**
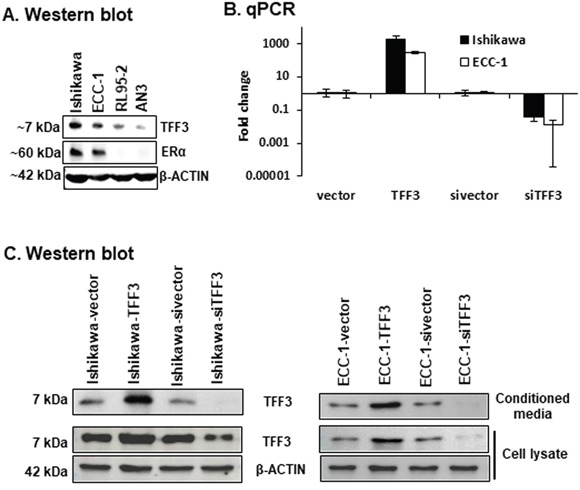
Generation of EC cells with stable forced or depleted expression of TFF3 **(A)** Western blot analysis was utilized to assess the levels of TFF3 and ERα protein in EC cells, namely Ishikawa, ECC-1, RL95-2, and AN3 cells. Soluble whole cell extracts were run on a SDS-PAGE and immunoblotted as described in materials and methods. β-ACTIN was used as input control for cell lysate. The predicted sizes of detected protein bands in kDa are shown on the *left side.* **(B)** *qPCR* analysis were used to evaluate the *mRNA* levels of *TFF3* genes in EC cells with either forced or depleted expression of TFF3. EC cells (vector or TFF3) were cultured in FM (10%FBS, standard media conditions as per ATCC propagation instructions) media. Either forced or depleted of *TFF3 expression* was achieved using stable transfection of TFF3 expression vector or *siRNA* directed to *TFF3 transcript* as described in materials and methods. *qPCR* analyses were performed as described in materials and methods. Statistical significance was assessed by using an unpaired two-tailed *Student's t test* (*P<0.05* was considered as significant) using GraphPad Prism5. **(C)** Western blot analysis was used to assess the levels of TFF3 in EC cells with either forced or depleted expression of TFF3. EC cells (vector or TFF3) were cultured in FM media. Either forced or depleted of *TFF3 expression* was achieved using stable transfection of TFF3 expression vector and *siRNA* directed to *TFF3 transcript* as described in materials and methods. Soluble whole cell extracts were run on a SDS-PAGE and immunoblotted as described in materials and methods. β-ACTIN was used as input control for cell lysate. The predictable sizes of detected protein bands in kDa are shown on the *left side.*

TFF3 has previously been demonstrated to stimulate the oncogenic behaviour of various carcinoma cells [18, 21, 23, 25–30]. Herein, we first analysed the four EC cell lines, namely Ishikawa, ECC-1, RL95-2 and AN3, to determine the endogenous protein levels of TFF3 and ERα (Figure 1A). Western blot analysis demonstrated that Ishikawa and ECC-1 cells expressed high endogenous levels of TFF3 protein, while RL95-2 and AN3 cells expressed relatively lower levels of TFF3 protein. In concordance with previously published reports [31], Ishikawa and ECC-1 cells exhibited high endogenous levels of ERα protein. However, ERα protein was not detected in RL95-2 and AN3 cells (Figure 1A). To define the cellular functionality of TFF3 in ERα positive EC cells, we generated Ishikawa and ECC-1 cells with stable forced expression or depletion of TFF3 as previously described [21, 32]. Pooled stable transfectants were used to minimize any effect of potential clonal selection. Forced expression or depletion of TFF3 in EC cells was confirmed using *qPCR* and western blot analysis (Figure 1B and 1C).

**Figure 2:**
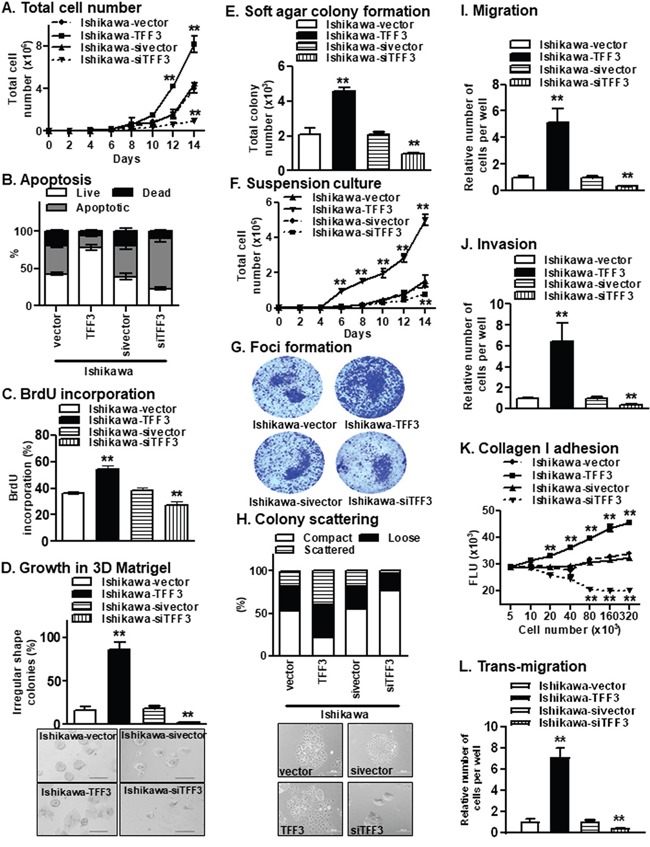
Forced expression of TFF3 in Ishikawa cells stimulates oncogenicity **(A)** Total cell number assay. Ishikawa cells with either forced or depleted expression of TFF3 were cultured in FM as described in materials and methods. **(B)** Apoptosis. Ishikawa cells with either forced or depleted expression of TFF3 were fixed and were stained for the assessment of early and late phase of apoptotic cell death using fluorescein isothiocyanate-conjugated annexin V (AV) and propidium iodide (PI), as described in materials and methods. **(C)** S-phase entry. The percentage of bromodeoxyuridine (BrdU)-positive cell nuclei relative to the total number of cell nuclei in Ishikawa cell with either forced or depleted expression of TFF3 was determined, as described in materials and methods. **(D)** Growth in Matrigel culture. Ishikawa cells were seeded in 2% Matrigel and irregular colonies formed were counted after incubation for fourteen days, as described in materials and methods. Representative images of colonies formed in Matrigel were presented below. **(E)** Soft agar colony formation. Ishikawa cells were seeded in 0.35% agarose and colonies (more than 50μm size) formed were counted after incubation for fourteen days, as described in materials and methods. **(F)** Growth in suspension culture. Ishikawa cells were seeded in low adherent plate and colonies (more than 50μm size) formed were counted after incubation for fourteen days, as described in materials and methods. **(G)** Foci formation. Ishikawa cells were seeded in six-well plate and stained with coomassie blue after incubation for fourteen days. Representative images of colonies formed were presented below. **(H)** Colony scattering assay. Distribution of compact, loose, and scattered colonies of Ishikawa cells with either forced or depleted expression of TFF3. Cells were plated at colony-forming conditions. One hundred colonies in each sample were categorized after scoring phase contrast images into three categories: compact (in which >90% of cells in a colony have cell-cell junctions), loose (in which 50–90% of cells form junctions), and scattered (in which <50% of cells form junctions), as described in materials and methods. Representative images of colonies formed were presented below. **(I)** Migration. Capacity of Ishikawa cells with either forced or depleted expression of TFF3 to migrate through membrane in trans-well chamber, as described in materials and methods. **(J)** Invasion. Capacity of Ishikawa cells with either forced or depleted expression of TFF3 to invade and migrate through Matrigel coated membrane in trans-well chamber, as described in materials and methods. **(K)** Collagen I adhesion. Capacity of Ishikawa cells with either forced or depleted expression of TFF3 to adhere to a Collagen I matrix, as described in materials and methods. **(L)** Trans-migration. Capacity of Ishikawa cells with either forced or depleted expression of TFF3 to transmigrate through a HMEC-1 layer, as described in materials and methods. Statistical significance was assessed by using an unpaired two-tailed *Student's t test* (*P<0.05* was considered as significant) using GraphPad Prism5. Columns or points are mean of triplicate experiments; bars, ±*SD*. **P < 0.001, *P < 0.05.

Forced expression of TFF3 increased Ishikawa total cell number, while depletion of TFF3 decreased Ishikawa total cell number over a period of 14 days when compared to the respective control cells (Figure 2A). Increased total cell number may be consequent to either a decrease in apoptosis and/or an increase in cell proliferation. Ishikawa-TFF3 cells exhibited reduced apoptotic cell death, while Ishikawa-siTFF3 cells exhibited increased apoptotic cell death upon serum deprivation as compared to their respective control cells (Figure 2B). In addition, Ishikawa-TFF3 cells showed increased S-phase entry, while Ishikawa-siTFF3 cells showed reduced S-phase entry as compared to their respective control cells in the BrdU incorporation assay, suggestive of TFF3-stimulated proliferation and cell cycle progression (Figure 2C). Furthermore, forced expression of TFF3 in Ishikawa cells increased 3D growth of the cells in Matrigel, with Ishikawa-TFF3 cells forming larger colonies of irregular morphologies in contrast to the regular spheroids formed by the Ishikawa-vector cells (Figure 2D). The depletion of TFF3 in Ishikawa cells, in contrast, decreased 3D growth of the cells in Matrigel, with Ishikawa-siTFF3 cells forming fewer and smaller colonies as compared to Ishikawa-sivector cells (Figure 2D). The forced expression and depletion of TFF3 in ECC-1 cells produced similar results in these assays (Supplementary Figure 1A-1C, 1F). We next determined the effect of forced expression of TFF3 on the expression of genes important for EC cell cycle progression and survival using *qPCR* (Table 1). The forced expression of TFF3 in the EC cells increased the *mRNA* levels of cyclins and cyclin-dependent kinases including *CCND1*, *CCNE1*, *CDK2*, *CDK4*, and decreased the *mRNA* levels of cyclin-dependent kinase inhibitor (*CDKN1B*) and *TP53*, concordant with the observed increase in cell cycle progression (Table 1). In contrast, the depletion of TFF3 in the EC cells exhibited the opposite effect on the expression of these cell cycle-related genes. Furthermore, the forced expression of TFF3 in EC cells increased the *mRNA* levels of anti-apoptotic *BCL2* and *BCL2L1*, and decreased the *mRNA* level of *CASP7*, concordant with the observed decrease in apoptosis (Table 1). In contrast, the depletion of TFF3 in the EC cells exhibited the opposite effect on the expression of these apoptosis-related genes. These observations suggest that TFF3 stimulates survival and proliferation of the EC cells through the modulation of genes involved in cell cycle progression and apoptosis.

**Table 1:** *qPCR* analysis for the *mRNA* levels of various genes associated with oncogenic progression of EC cells with either forced or depleted expression of TFF3

Gene	Ishikawa				ECC1			
	Forced expression of TFF3		Depleted expression of TFF3		Forced expression of TFF3		Depleted expression of TFF3	
	Fold change	*p value*	Fold change	*p value*	Fold change	*p value*	Fold change	*p value*
*CCND1*	5.80	1.40E-02	0.21	2.70E-02	6.08	1.92E-02	0.32	2.64E-02
*ATM*	2.52	4.58E-03	0.67	1.71 E-02	3.23	7.68E-03	0.39	3.63E-03
*CCNE1*	3.43	6.34E-04	0.17	1.87E-02	2.84	6.14E-03	0.11	1.03E-02
*CDK2*	2.08	3.01 E-02	0.20	1.73E-02	3.11	3.12E-03	0.15	1.73E-02
*CDK4*	2.21	3.61 E-02	0.07	7.28E-03	2.12	2.09E-02	0.03	1.00E-02
*CDKN1A*	0.84	4.63E-02	0.26	1.78E-02	1.46	5.22E-03	0.75	9.12E-05
*CDKN2A*	1.53	1.23E-02	0.44	4.68E-02	3.26	7.92E-04	0.12	6.93E-03
*CHEK2*	2.60	2.30E-02	0.11	1.62E-03	2.91	3.35E-06	0.34	9.00E-04
*MDM2*	9.45	3.87E-03	0.11	2.39E-02	6.49	7.34E-04	0.07	2.10E-03
*S100A4*	1.76	1.75E-02	0.59	1.63E-02	1.58	5.64E-03	0.47	6.85E-03
*TP53*	0.07	2.03E-02	9.19	9.69E-03	0.04	2.10E-03	11.38	1.21E-03
*CDKN1B*	0.15	2.14E-02	3.67	6.45E-04	0.11	1.03E-03	4.82	1.20E-03
*APAF1*	2.39	1.15E-02	0.10	1.64E-03	3.21	4.24E-02	0.04	1.80E-02
*BCLAF1*	1.06	2.08E-02	0.29	2.29E-03	1.12	2.22E-02	0.32	2.51E-02
*BAK1*	1.18	9.52E-02	0.30	2.71 E-04	1.26	5.69E-03	0.72	8.01E-03
*BAD*	8.60	4.96E-03	0.17	2.17E-03	11.43	1.12E-03	0.05	1.55E-02
*BCL2*	18.25	1.33E-03	0.17	1.12E-02	15.94	4.41 E-03	0.09	5.58E-03
*BCL2L1*	3.86	1.90E-02	0.14	7.63E-03	5.63	1.02E-02	0.07	6.13E-02
*CASP7*	0.21	1.11 E-02	8.20	1.53E-02	0.13	3.03E-02	16.03	7.98E-03
*TERT*	2.86	1.54E-02	0.51	4.21 E-02	3.67	2.17E-03	0.01	2.13E-03
*TNFRSF10B*	4.52	8.66E-03	0.37	1.98E-02	6.04	1.46E-03	0.29	4.08E-03
*MET*	2.18	1.10E-02	0.38	3.20E-05	3.46	2.24E-03	0.09	1.68E-02
*NME1*	1.03	1.71 E-02	0.89	2.57E-02	1.94	7.84E-04	0.05	1.51E-03
*PLAU*	4.90	8.57E-05	0.23	2.30E-02	5.02	1.73E-03	0.34	2.49E-02
*SERPINE1*	5.56	3.00E-03	0.13	2.29E-02	7.02	1.64E-02	0.11	2.56E-02
*TWIST1*	2.17	7.95E-03	0.23	4.06E-02	3.09	7.46E-03	0.09	5.84E-04
*VIM*	7.64	9.01 E-03	0.12	3.66E-02	10.37	4.73E-02	0.26	3.85E-03
*FN1*	3.30	3.51 E-03	0.21	3.44E-02	6.39	1.84E-03	0.11	2.75E-03
*OCLN*	0.22	3.61 E-03	4.72	4.01 E-03	0.01	4.38E-03	7.38	2.83E-02
*CTNNA1*	0.88	4.01 E-02	0.30	2.88E-02	1.84	4.94E-03	0.25	3.76E-02
*CTNNB1*	0.81	3.70E-02	0.28	2.97E-02	1.55	4.84E-02	0.22	3.47E-03
*CTNND1*	1.59	3.87E-02	0.28	1.70E-02	3.92	3.82E-02	0.36	3.75E-03
*CDH1*	0.06	1.93E-02	5.03	1.74E-03	0.04	3.95E-04	13.95	4.74E-02
*CD44*	0.77	1.61 E-02	0.40	2.70E-02	1.26	1.85E-02	0.56	4.33E-02
*SNAIL*	1.77	1.03E-02	0.12	3.01 E-02	2.04	3.85E-02	0.25	1.85E-02
*CDH2*	5.43	7.29E-03	0.27	1.11 E-02	8.03	3.83E-04	0.14	3.75E-03
*ALDH1*	7.66	7.00E-03	0.08	7.88E-03	11.48	2.85E-02	0.09	4.39E-01

Change in gene expression is expressed as fold difference, respectively. Fold change values are representative of three independent biological experiments. To compensate for potential differences between markers, the relative expression was computed, based on the efficiency (E), normalized by a panel of housekeeping genes, *β-ACTIN, HPRT*, and *GAPDH*.

A characteristic of oncogenically transformed cells is the capacity for anchorage-independent growth, which is studied using the soft agar colony formation, suspension culture and foci formation assays. Ishikawa-TFF3 cells exhibited higher anchorage-independent cell growth, as indicated by increased colony formation in soft agar (Figure 2E), increased cell growth in suspension culture (Figure 2F) and increased foci formation (Figure 2G), as compared to Ishikawa-vector cells. In contrast, depletion of TFF3 decreased anchorage-independent cell growth of Ishikawa cells in the respective assays (Figure 2E-2G). Similar directional changes in anchorage-independent growth were observed in ECC-1 cells with either forced or depleted expression of TFF3 (Supplementary Figure 1D-1E). Hence, TFF3 promotes increased oncogenicity of EC cells.

We next determined the effect of TFF3 on the migratory and invasive properties of EC cells. In colony scattering assay, Ishikawa-TFF3 cells formed a higher percentage of loose and scattered colonies, and a lower percentage of compact colonies as compared to Ishikawa-vector cells (Figure 2H). On the other hand, Ishikawa-siTFF3 cells formed a higher percentage of compact colonies, a lower percentage of loose colonies and an even lower percentage of scattered colonies, as compared to Ishikawa-sivector cells (Figure 2H). As compared to their respective control cells, Ishikawa-TFF3 cells exhibited more mesenchymal characteristics including an elongated morphology with multiple cellular protrusions and reduced cell-cell contact, while Ishikawa-siTFF3 cells exhibited more epithelial characteristics forming defined grouped colonies with copious cell-cell contact (Figure 2H). In addition, forced expression of TFF3 significantly increased cell migration, while depletion of TFF3 significantly reduced cell migration of Ishikawa cells in the Transwell assay (Figure 2I). Ishikawa-TFF3 cells also demonstrated significantly increased invasion, while Ishikawa-siTFF3 cells showed significantly decreased invasion through the Matrigel-coated Transwell, as compared to their respective control cells (Figure 2J). As the metastatic process also involves the adhesion of tumour cells to, and invasion through, the surrounding extracellular matrix [21], we investigated the effect of TFF3 on the ability of EC cells to adhere to collagen I, which is a major component of the stromal matrix. The forced expression of TFF3 increased the adhesion of Ishikawa cells to collagen I, while depletion of TFF3 decreased the adhesion of Ishikawa cells to collagen I (Figure 2K). Furthermore, tumour cells adhere to and migrate through endothelial cells lining blood vessels during metastasis [21]. Ishikawa-TFF3 cells showed increased transmigration through the endothelial cell barrier as compared to Ishikawa-vector cells (Figure 2L). In contrast, Ishikawa-siTFF3 cells showed decreased transmigration through the endothelial cell barrier as compared to Ishikawa-sivector cells (Figure 2L). Similar directional changes in invasive and migratory properties were observed in ECC-1 cells with either forced or depleted expression of TFF3 (Supplementary Figure 1H-1J). We further determined the effect of forced expression of TFF3 on the expression of epithelial mesenchymal transition (EMT)-related genes using *qPCR* (Table 1). The forced expression of TFF3 in the EC cells increased the *mRNA* levels of mesenchymal gene markers including *TWIST1*, *VIM*, *FN1* and *SNAIL*, and decreased the mRNA levels of epithelial gene markers including *OCLN* and *CDH1*, concordant with the observed TFF3-mediated invasion and migration of EC cells. In contrast, the depletion of TFF3 in the EC cells exhibited the opposite effect on the expression of these EMT-related genes. Furthermore, the forced expression of TFF3 increased the *mRNA* levels of metastasis-associated genes *MET* and *PLAU*, while the depletion of TFF3 exhibited a decrease in the expression of these genes in the EC cells. Hence, TFF3 increases the EMT of EC cells through the modulation of epithelial, mesenchymal and metastasis-related gene markers.

### TFF3 enhances tumorigenicity of EC cells *in vivo*

**Figure 3:**
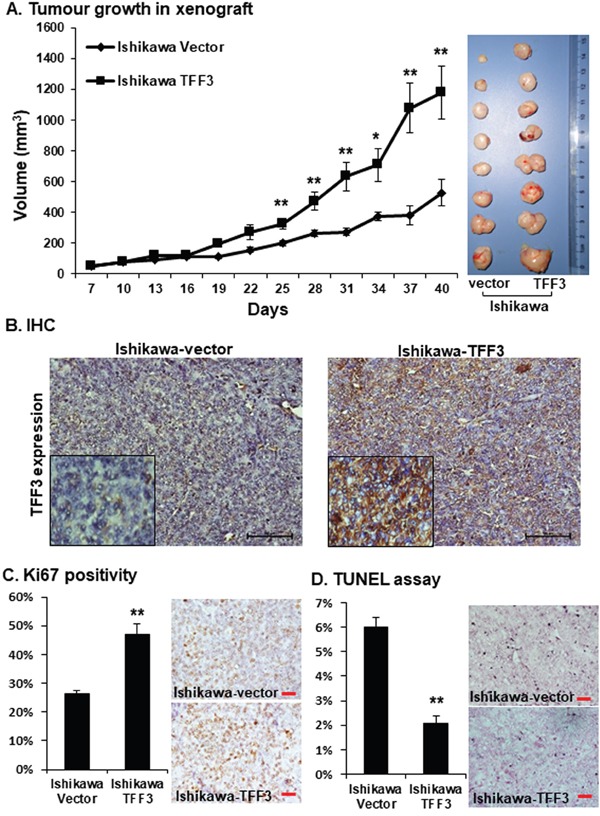
Forced expression of TFF3 in Ishikawa cell enhances tumour formation in immunodeficient mice Ishikawa cells with forced expression of TFF3 and their vector control cells in Matrigel were injected SC into immunodeficient mice and allow to grow for defined period as described in materials and methods. **(A)** Tumour volume in relation to the day of surgery shown. Resected tumour masses generated from Ishikawa-TFF3 and Ishikawa-vector cells in mice are shown on the *right side*. **(B)** TFF3 expression was assessed in resected tumour masses generated from Ishikawa-TFF3 and Ishikawa-vector cells in mice using immunohistochemistry (IHC) as described in materials and methods. **(C)** Cell proliferation was assessed using Ki67 positivity staining as described in materials and methods. **(D)** Apoptosis was measured by TUNEL labelling as described in materials and methods. Statistical significance was assessed by using an unpaired two-tailed *Student's t test* (*P<0.05* was considered as significant) using GraphPad Prism5. Columns or points are mean of triplicate experiments; bars, ±*SD*. **P < 0.001, *P < 0.05.

To determine whether TFF3 enhances growth of EC cells *in vivo*, we injected Ishikawa-vector and Ishikawa-TFF3 cells subcutaneously into the dorsal flanks of female nude mice. Palpable tumours derived from each cell line were formed within a week, and tumour sizes were measured every 3 days. The Ishikawa-TFF3 cell-derived tumours grew at a significantly faster rate than the Ishikawa-vector cell-derived tumours from day 25 onwards, resulting in a larger tumour mass on day 40 (Figure 3A). Next, we performed immunohistochemistry (IHC) analysis for TFF3 on resected tumours generated using Ishikawa-vector or Ishikawa-TFF3 cells. Tumours generated from Ishikawa-TFF3 cells exhibited markedly increased expression of TFF3 compared to tumours generated from Ishikawa-vector cells (Figure 3B) indicative of phenotypic retention. The percentage of the TFF3-positive cell population in Ishikawa-TFF3 cell-derived tumours was significantly higher (84.63±5.82% vs 14.86±3.08%, *p value* <0.001) as compared to Ishikawa-vector cell-derived tumours. We further determined the effect of TFF3 on tumour cell proliferation and apoptosis *in vivo* using Ki67 staining and TUNEL assay respectively. The percentage of Ki67-positive cell population in Ishikawa-TFF3 cells-derived tumours was significantly higher as compared to Ishikawa-vector cells-derived tumours (Figure 3C). In contrast, the tumours generated from Ishikawa-TFF3 cells contained significantly less apoptotic nuclei than tumours formed from Ishikawa-vector cells in the TUNEL assay (Figure 3D). Therefore, TFF3 promotes xenograft growth of Ishikawa cells by increasing tumour cell proliferation and reducing tumour cell apoptosis.

### High TFF3 expression is associated with EC and myometrial invasion

**Figure 4:**
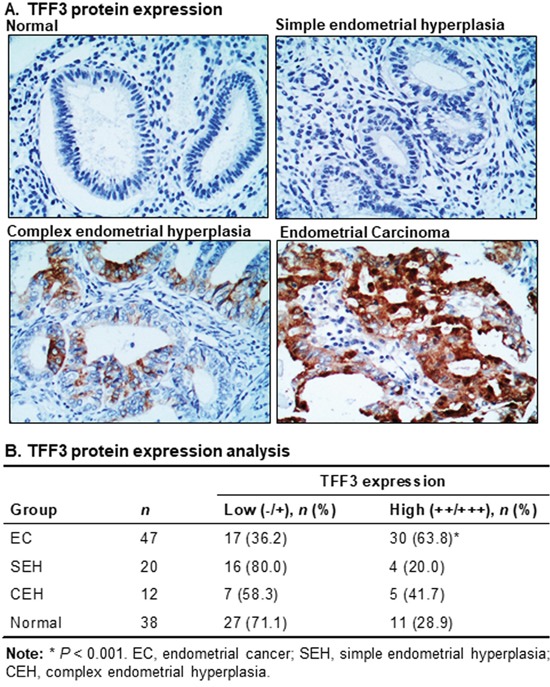
High TFF3 expression is observed in EC patient tissue specimens **(A)** TFF3 expression was detected using immunohistochemistry (IHC) in normal (endometrium), simple endometrial hyperplasia (SEH), complex endometrial hyperplasia (CEH), and EC tissue specimens. TFF3 was predominantly localized in the cytoplasm of carcinoma cells in EC tissue specimens. Representative images of TFF3 expression in normal, SEH, CEH, and EC tissue specimens were captured under X200 magnification (*above*). **(B)** TFF3 expression was analysed in normal, SEH, CEH, and EC tissue specimens using IHC. Statistical significance was assessed by using an unpaired two-tailed *Student's t test* (*P<0.05* was considered as significant) using GraphPad Prism5.

To determine the potential clinical relevance of TFF3 expression in EC patients, we analyzed the expression levels of TFF3 protein in normal endometrial tissues, endometrial hyperplasia and EC using IHC. High TFF3 expression was observed in EC specimens, while absent or lower TFF3 expression was detected in the normal endometrium and hyperplastic tissues (Figure 4A). The percentage of EC patients with high TFF3 expression was significantly higher than the percentage of EC patients with absent or low TFF3 expression (Figure 4B). In contrast, in cohorts with normal endometrium, simple endometrial hyperplasia (SEH) and complex endometrial hyperplasia (CEH), the percentage of cases with absent/low TFF3 expression was higher as compared to the percentage of cases with high TFF3 expression, although the differences were not statistically significant (Figure 4B). TFF3 expression in EC was found to be significantly correlated with enhanced myometrial invasion (Table 2). There was also a tendency for TFF3 expression to be associated with higher FIGO grade and cervical involvement although statistical significance was not reached.

**Table 2:** Association of TFF3 expression with clinicopathological parameters from endometrial cancer patients

Parameter	*n*	TFF3 expression(++/+++), *n* (%)	*p* value
Age (years)			
< 60	40	25 (62.5)	0.650
≥ 60	7	5 (71.4)	
Menopausal status		4	
Premenopausal	12	7 (58.3)	0.646
Postmenopausal	35	23 (65.7)	
FIGO stage			
I + II	42	25 (59.5)	0.075
III+IV	5	5 (100.0)	
FIGO grade			
1	21	12 (57.1)	0.391
2 + 3	26	18 (69.2)	
Lymph node metastasis			
–	44	27 (61.4)	0.178
+	3	3 (100.0)	
Myometrial invasion			
No	6	0(0)	**0.001**
Yes	41	30 (73.2)	
Cervical involvement			
Negative	42	25 (59.5)	0.075
Positive	5	5 (100.0)	
Ovarian metastasis			
Negative	44	27 (61.4)	0.178
Positive	3	3(100)	
Estrogen receptor (ER)			
–	13	8 (61.5)	0.840
+	34	22 (64.7)	
Progesterone receptor (PR)			
–	13	8 (61.5)	0.840
+	34	22 (64.7)	

Note: Cohort size (N)=47; statistical analyses of patient's samples was performed using SPSS software (version 13.0; SPSS, Chicago, IL). *P<0.05* was considered as significant. Estrogen or progesterone receptor positive required at least 10% staining nuclei.

### Acute TAM exposure increases TFF3 expression and enhances cell viability of ER+ EC cells

**Figure 5:**
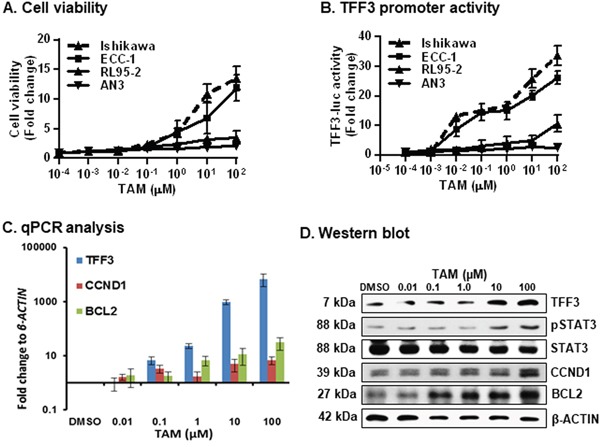
TAM exposure of ER+ EC cells stimulates cell viability, increased promoter activity of *TFF3*, expression of TFF3/CCND1/BCL2 and STAT3 activity **(A)** Cell viability was measured in EC cells after exposure to increased doses of TAM. Cell viability was measured using Alamar Blue as described in materials and methods. **(B)** *TFF3 promoter* activity was measured in EC cells after exposure to increasing doses of TAM. Universal controls (scrambled oligo) were used for transfection control as described in materials and methods. The luciferase assay was performed as described in materials and methods. **(C)** *qPCR* analysis was used to evaluate the *mRNA* levels of *TFF3*, *CCND1*, and *BCL2* gene in Ishikawa cells after exposure to increasing doses of TAM. *qPCR* analysis were performed as described in materials and methods. Change in gene expression is expressed as fold difference relative to *mRNA* levels of *β-ACTIN*. Fold change values are representative of three independent biological experiments. **(D)** Western blot analysis was used to assess the levels of TFF3, pSTAT3, STAT3, BCL2, and CCND1 in Ishikawa cells after exposure to increased doses of TAM. Soluble whole cell extracts were run on a SDS-PAGE and immunoblotted as described in materials and methods. β-ACTIN was used as input control for cell lysate. The predicted sizes of detected protein bands in kDa are shown on the *left side.* Statistical significance was assessed by using an unpaired two-tailed *Student's t test* (*P<0.05* was considered as significant) using GraphPad Prism5. Columns or points are mean of triplicate experiments; bars, ±*SD*. **P < 0.001, *P < 0.05.

To determine the potential association between TAM-driven agonistic activities and TFF3 expression in EC cells, we first utilized cell viability analysis to determine the effect of acute TAM exposure (48 hour) in Ishikawa, ECC-1, RL95-2 and AN3 cells. Acute TAM exposure resulted in a dose-dependent increase in cell viability of Ishikawa and ECC-1 cells (Figure 5A); whereas, ER-negative and low TFF3-expressing RL95-2 and AN3 cells exposed to TAM exhibited only marginal increases in cell viability at higher doses of TAM. We also verified that TAM was acting as an ERα agonist over the utilized dose range of TAM by use of an oestrogen response element (ERE) reporter assay in Ishikawa cells (Supplementary Figure 2A). Higher doses of TAM has been reported to exhibit cytotoxic effects in EC cells [33]. We therefore verified that TAM was not cytotoxic in Ishikawa cells over the utilized dose range (5μM) of TAM as indicated by use of ApoTox-Glo^™^ Triplex Assay, Promega (Supplementary Figure 2B).

We next assessed *TFF3 promoter activity* using a *TFF3-luciferase (-luc)* reporter construct in Ishikawa, ECC-1, RL95-2 and AN3 cells after exposure to TAM. The *TFF3-luc reporter* construct contains a fragment of the *TFF3 gene promoter* (−700 to +50 bp) as described in the materials and methods section. On exposure to increasing concentrations of TAM, Ishikawa and ECC-1 cells exhibited dose-dependent increases in *TFF3 promoter activity* (Figure 5B); whereas, RL95-2 and AN3 cells exposed to TAM exhibited only marginal increases in *TFF3 promoter* activity at higher doses of TAM. As TFF3 has previously been demonstrated to increase STAT3 activity and expression of CCND1 and BCL2 [21], we next utilized *qPCR* and western blot analysis to examine the *mRNA* and protein levels of TFF3, CCND1, BCL2 and pSTAT3 in Ishikawa cells after exposure to TAM. Consistent with above findings, Ishikawa cells exposed to TAM exhibited dose-dependent increases in the *mRNA* levels of *TFF3, CCND1* and *BCL2* (Figure 5C); and dose dependent increase in protein levels of TFF3, CCND1, BCL2 and pSTAT3. No significant change was observed in total STAT3 protein levels in Ishikawa cells after exposure to TAM (Figure 5D). Thus, acute TAM exposure consequently increased *TFF3* expression and enhanced cell viability of ER+ Ishikawa and ECC-1 cells.

### Chronic exposure of ER+ EC cells to TAM stimulates growth in 3D Matrigel culture, anchorage independent cell growth, and cell invasion through TFF3

**Figure 6:**
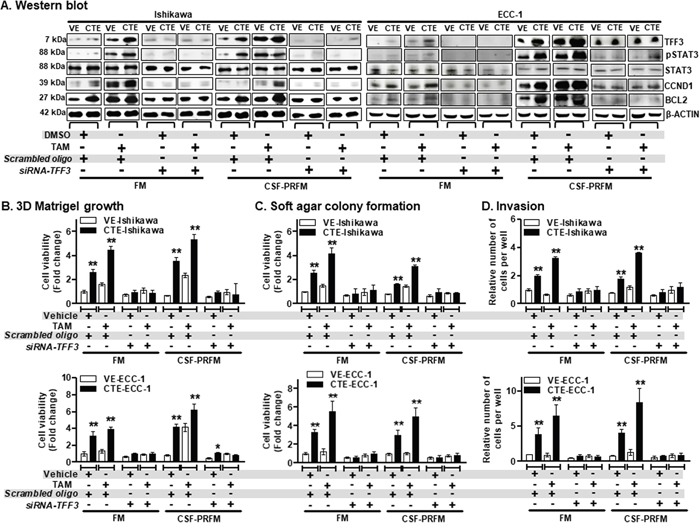
TAM-stimulated oncogenic behaviour of ER+ EC cells is significantly abrogated after depletion of TFF3 expression **(A)** Western blot analysis was used to assess the levels of TFF3, pSTAT3, STAT3, CCND1, and BCL2 protein in EC cells, namely Ishikawa and ECC-1 with chronic TAM exposed (CTE) or their vehicle exposed (VE). EC cells (VE or CTE) were cultured in FM or CSF-PRFM. 5μM TAM was treat cells. Depletion of *TFF3* expression was achieved using transient-transfection of *small interfering* (*si*)-*RNA* directed to *TFF3 transcript* as described in materials and methods. Soluble whole cell extracts were run on a SDS-PAGE and immunoblotted as described in materials and methods. β-ACTIN was used as input control for cell lysate. The predicted sizes of detected protein bands in kDa are shown on the *left side.* **(B)** 3D Matrigel growth, **(C)** soft agar colony formation and **(D)** trans-well cell invasion of EC cells with CTE or VE. EC cells (VE or CTE) were cultured in FM or CSF-PRFM. 5μM TAM was used to treat cells. Depletion of *TFF3* expression was achieved using transient-transfection of *small interfering* (*si*)-*RNA* directed to *TFF3 transcript* as described in materials and methods. Cell viability of EC cells (CTE or VE) measured using AlamarBlue® viability assay as described in materials and methods. FM were 10%FBS, standard media conditions as per ATCC propagation instructions; and CSF-PRFM were charcoal stripped 10% FBS, phenol-red free media. Statistical significance was assessed by using an unpaired two-tailed *Student's t test* (*P<0.05* was considered as significant) using GraphPad Prism5. Columns are mean of triplicate experiments; bars, ±*SD*. **P < 0.001, *P < 0.05.

To determine the functional role of TFF3 in TAM-stimulated agonistic activities in EC cells, we generated an *in vitro* model of Ishikawa and ECC-1 cell lines with chronic (18 weeks) TAM exposure (CTE) or vehicle (DMSO) exposure (VE) as described in materials and methods. Cell lines were designated as CTE-Ishikawa/VE-Ishikawa and CTE-ECC-1/VE-ECC-1 cells. Using western blot analysis we first determined that CTE-Ishikawa cells exhibited higher basal TFF3 protein levels compared to VE-Ishikawa cells when cultured in 10%FBS-media (FM) or charcoal-stripped 10%FBS phenol-red free media (CSF-PRFM) (Figure 6A). On exposure to TAM, both CTE-Ishikawa and VE-Ishikawa cells exhibited augmented levels of TFF3 protein compared to the respective vehicle (DMSO) exposed cells (Figure 6A). Nevertheless, CTE-Ishikawa cells maintained higher protein levels of TFF3 compared to VE-Ishikawa cells in the presence of TAM (Figure 6A). Moreover, CTE-Ishikawa cells also exhibited increased pSTAT3 activity and higher basal protein levels of CCND1 and BCL2 compared to VE-Ishikawa cells when cultured in FM or CSF-PRFM (Figure 6A). The total protein levels of STAT3 were not significantly altered in Ishikawa cells with either CTE- or VE- when cultured in FM or CSF-PRFM. On exposure to TAM, both CTE-Ishikawa and VE-Ishikawa cells exhibited augmented levels of pSTAT3 activity and CCND1 and BCL2 protein compared to the respective vehicle (DMSO) exposed cells (Figure 6A). Consistently, CTE-Ishikawa cells maintained increased pSTAT3 activity and higher protein levels of CCND1 and BCL2 compared to VE-Ishikawa cells in the presence of TAM (Figure 6A). The total protein levels of STAT3 were not significantly altered in Ishikawa cells with either CTE- or VE- when exposed to TAM or DMSO. Hence, CTE-Ishikawa cells exhibited higher TFF3 protein levels, and corresponding higher levels of pSTAT3, CCND1 and BCL2 as compared to VE-Ishikawa cells, both basally and with acute TAM exposure.

Next, we employed validated *siRNA* [34] to reduce expression of TFF3 in both CTE-Ishikawa and VE-Ishikawa cells when cultured in FM or CSF-PRFM. *siRNA*-mediated depletion of TFF3 protein in both CTE-Ishikawa or VE-Ishikawa cells markedly decreased basal protein levels of TFF3 compared to their control cells (Figure 6A). Depletion of TFF3 expression in both CTE-Ishikawa and VE-Ishikawa cells also resulted in decreased protein levels of CCND1 and BCL2, and pSTAT3 activity as compared to their control cells (Figure 6A). Moreover, TAM-stimulated protein levels of TFF3, CCND1, and BCL2; and pSTAT3 activity were abrogated after depletion of TFF3 expression in both CTE-Ishikawa and VE-Ishikawa cells (Figure 6A). CTE-Ishikawa and VE-Ishikawa cells cultured in CSF-PRFM exhibited augmented protein levels of TFF3, CCND1, and BCL2; and pSTAT3 activity compared to cells cultured in FM (Figure 6A). Similar directional changes in protein levels of TFF3, CCND1, BCL2, and pSTAT3 activity were observed in acute TAM or DMSO treated CTE- or VE- ECC-1 cells, and upon TFF3 depletion in these cells under the same conditions (Figure 6A).

To determine the functional consequences of enhanced TFF3 expression in TAM-treated EC cells, we next examined 3D Matrigel growth, anchorage independent growth and invasiveness of CTE-Ishikawa and VE-Ishikawa cells when cultured in FM or CSF-PRFM after exposure to TAM or DMSO. In 3D Matrigel culture, CTE-Ishikawa cells exhibited increased cell viability compared to VE-Ishikawa cells when cultured in FM or CSF-PRFM (Figure 6B). On exposure to TAM, both CTE-Ishikawa and VE-Ishikawa cells exhibited increased 3D growth as compared to the respective vehicle-exposed cells (Figure 6B). CTE-Ishikawa cells maintained significantly increased 3D growth compared to VE-Ishikawa cells in the presence of TAM (Figure 6B). *siRNA*-mediated depletion of TFF3, decreased both basal and TAM-stimulated 3D growth of CTE-Ishikawa or VE-Ishikawa cells (Figure 6B). Notably, the stimulatory effect of TAM exposure on 3D growth was largely abrogated by TFF3 depletion in Ishikawa cells in either CTE- or VE-cells. In soft agar colony formation and foci formation assays, CTE-Ishikawa cells exhibited enhanced anchorage independent growth capacity compared to VE-Ishikawa cells when cultured in FM or CSF-PRFM (Figure 6C and Supplementary Figure 2C). On exposure to TAM, both CTE-Ishikawa and VE-Ishikawa cells exhibited enhanced soft agar colony formation and foci formation compared to vehicle-exposed cells, although CTE-Ishikawa cells still maintained significantly higher anchorage-independent growth capacity than VE-Ishikawa cells (Figure 6C and Supplementary Figure 2C). Depletion of TFF3 in CTE-Ishikawa or VE-Ishikawa cells reduced both basal and TAM-stimulated soft agar colony formation and foci formation (Figure 6C and Supplementary Figure 2C). In particular, the stimulatory effect of TAM exposure on anchorage-independent growth was abrogated after depletion of TFF3 expression in either CTE- or VE- Ishikawa cells. In transwell invasion assays, CTE-Ishikawa cells exhibited increased cell invasion capacity through Matrigel compared to VE-Ishikawa cells when cultured in FM or CSF-PRFM (Figure 6D). On exposure to TAM, both CTE-Ishikawa and VE-Ishikawa cells demonstrated enhanced cell invasion capacity through Matrigel compared to vehicle-exposed cells, with CTE-Ishikawa cells maintaining a significantly higher invasive capacity than VE-Ishikawa cells (Figure 6D). *siRNA-mediated* depletion of TFF3 expression in CTE-Ishikawa or VE-Ishikawa cells largely prevented cell invasion through Matrigel (Figure 6D). Particularly, the stimulatory effect of TAM on cell invasion was abrogated after depletion of TFF3 expression in CTE-Ishikawa and VE-Ishikawa cells. ECC-1 cells with either CTE- or VE- exhibited similar directional changes in 3D Matrigel growth, anchorage independent growth and cell invasion on exposure to TAM or DMSO, and upon the depletion of TFF3 in these cells under the same conditions (Figure 6B-6D). Thus, chronic TAM exposure stimulates growth, oncogenicity and invasiveness of ER+ EC cells in a TFF3-dependent manner.

### TAM-stimulated increase in TFF3 expression occurs through hypomethylation of the *TFF3 promoter* and increased transcriptional activity of *c-JUN/SP1*

**Figure 7:**
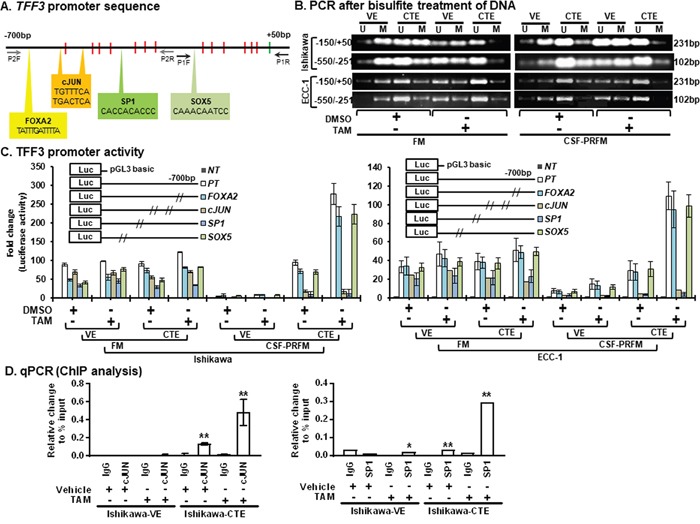
TAM stimulates hypomethylation in the *TFF3 promoter* and modulates *TFF3 transcription* through c-JUN/SP1 **(A)** *TFF3 promoter* sequence (from -700 to +50bp) located on chromosome 21q22.3 (21:42316053-42314804). Thirteen CpG islands are positioned in the *TFF3 promoter* sequence, highlighted in red colour. Four binding sites for transcription factors, *FOXA2* (*TATTTGATTTTA*, -667 to -656bp), *c-JUN* (*TGTTTCA*, -532 to -525bp; *TGACTCA*, -498 to -491bp), *SP1* (*CACCACACCC*, -379 to -369bp), and *SOX5* (*CAAACAATCC*, -119 to -110bp) were identified in the *TFF3 promoter* sequence, highlighted in bold font. Starting site, ATG, highlighted in green colour. Primer sites highlighted in black (P1) and grey (P2) colour. **(B)** *PCR* for bisulfite treated *DNA*. Total *DNA* was extracted from cells and treated with bisulfite as described in materials and methods. Sequence of unmethylated (U) and methylated (M) specific primers described in materials and methods. Primer specific sequence amplified on *TFF3 promoter* mentioned *left side*. The sizes of detected amplified product in *base pair* (bp) are shown on the *right side.* EC cells (VE or CTE) were cultured in FM or CSF-PRFM. 5μM TAM was used to treat cells. **(C)** *TFF3 promoter* activity in EC cells. EC cells (VE or CTE) were cultured in FM or CSF-PRFM. 5μM TAM was used to treat cells. The luciferase assay was performed as described in Materials and Methods. *NT*, negative control (*pGL3 basic construct*); *PT*, positive control (*pGL3-TFF3 construct*); *pGL3-TFF3 construct* with altered sequence of *FOXA, c-JUN, SP1*, or *SOX5* binding site (mentioned in Figure 2A), respectively. Construct information is described in methodology section. **(D)** ChIP analysis in EC cells. EC cells (VE or CTE) were cultured in FM or CSF-PRFM. 5μM TAM was used to treat cells. ChIP assay was carried out using IgG (control) or SP1 antibody and c-JUN antibody and binding of SP1 and c-JUN was assessed using *q-PCR* as described in the methodology section. For each PCR, enrichment represent relative to the percentage of Input, error bars represent *±SD*. FM were 10%FBS, standard media conditions as per ATCC propagation instructions; and CSF-PRFM were charcoal striped 10% FBS, phenol-red free media. Statistical significance was assessed by using an unpaired two-tailed *Student's t test*. Columns are mean of triplicate experiments; bars, ±*SD*. **P < 0.001, *P < 0.05.

To understand the mechanism of enhanced TFF3 expression in EC cells after chronic exposure to TAM, we examined the methylation status of the *proximal TFF3 promoter* in CTE-Ishikawa and VE-Ishikawa cells using methylation-specific PCR after genomic bisulfite modification as described in materials and methods. The promoter sequence of *TFF3* from -700bp to +50bp contains 13 CpG’s, out of which 7 CpG's are located between -550bp to -251bp and 6 CpG's are located between -150bp to +50bp. Two set of primers were designed for methylated or un-methylated sequence from -150bp to +50bp (P1) and -550bp to -251bp (P2) as described in materials and methods (Figure 7A). Using PCR analysis, both P1 & P2 PCR product demonstrated that the *TFF3 promoter* sequence was hypomethylated in CTE-Ishikawa cells as compared to VE-Ishikawa cells when cultured in either FM or CSF-PRFM (Figure 7B). Upon acute exposure to TAM, both CTE-Ishikawa cells and VE-Ishikawa cells showed decreased *TFF3 promoter* methylation (Figure 7B). Notably, TAM exposure rendered the *TFF3 promoter* in CTE-Ishikawa cells to be almost completely non-methylated as compared to vehicle treated cells (Figure 7B). Hence, cells cultured in CSF-PRFM exhibited hypomethylation of the *TFF3 promoter* compared to cells cultured in FM media (Figure 7B).

We next sought to determine the transcription factors that bind the *TFF3 promoter* to regulate TFF3 transcription in EC cells with acute and/or chronic TAM exposure. Using the online Transcriptional Regulatory Element Database (TRED) (https://cb.utdallas.edu/cgi-bin/TRED/tred.cgi?process=home), we identified four transcription factor binding sites in the *TFF3 promoter* sequence between -700bp to +50bp namely FOXA2, c-JUN, SP1, and SOX5 (Figure 7A). FOXA2, c-JUN, and SP1 transcription factor binding sites are found to be located in -550bp to -251bp (P2) of the *TFF3 promoter* sequence. Only the SOX5 transcription factor binding site is located in -150bp to +50bp (P1) of the *TFF3 promoter* sequence. To determine whether FOXA2, c-JUN, SP1, and/or SOX5 is/are involved in TAM-stimulated TFF3 transcription in EC cells, the specific TF binding sequences were serially mutated in the *TFF3-luc reporter construct* and transfected into CTE-Ishikawa and VE-Ishikawa cells for subsequent detection of luciferase activity. As represented in Figure 7C, both CTE-Ishikawa and VE-Ishikawa cells transfected with each mutant *TFF3-luc* construct resulted in decreased luciferase activities compared to wildtype *TFF3-luc* (positive control) construct. Amongst the mutant *TFF3-luc* constructs, SP1 site mutated *TFF3-luc construct* demonstrated the lowest luciferase activities in CTE-Ishikawa cells under FM conditions (Figure 7C). Furthermore, c-JUN or SP1 site mutated *TFF3-luc construct* showed lowest and/or marginal luciferase activities in CTE-Ishikawa cells under CSF-PRFM conditions. Under FM conditions, no significant changes in *TFF3 promoter* luciferase activities were observed in CTE- and VE- Ishikawa cells, or upon acute exposure of these cells to TAM. Under CSF-PRFM conditions, CTE-Ishikawa cells exhibited markedly higher wildtype *TFF3 promoter* luciferase activities as compared to VE-Ishikawa cells. Notably, mutations in the SP1 or c-JUN transcription factor binding sites of the *TFF3 promoter* abrogated the increased transcriptional activity at the *TFF3 promoter* of the CTE-Ishikawa cells. Upon TAM exposure under CSF-PRFM conditions, the wildtype *TFF3 promoter* luciferase activities were significantly increased in both the CTE-Ishikawa and VE-Ishikawa cells. Similarly, mutation of the SP1 or c-JUN transcription factor binding sites of the *TFF3 promoter* prevented the TAM-stimulated increase in *TFF3 promoter* transcriptional activity of both the CTE-Ishikawa and VE-Ishikawa cells. Similar directional changes in methylation status and *TFF3 promoter* luciferase activities was observed in ECC-1 cells with either CTE- or VE- under the same conditions (Figure 7B and 7C). To further determine the regulation of TFF3 by c-JUN and/or SP1, we performed chromatin immunoprecipitation (ChIP) analysis. Both c-JUN and SP1 binding sites of the *TFF3 promoter* interacted with c-JUN and SP1 proteins respectively in CTE-Ishikawa cells. Upon TAM exposure, c-JUN and SP1 proteins demonstrated significantly increased interaction with their respective binding sites on the *TFF3 promoter* in CTE-Ishikawa cells (Figure 7D). These observations suggest that SP1 and c-JUN transcription factors are involved in increased TFF3 transcription in acute or chronic TAM-exposed EC cells.

### TAM-stimulated TFF3 upregulation and 3D growth of EC cells occurs in a *c-JUN/SP1*-dependent manner

**Figure 8:**
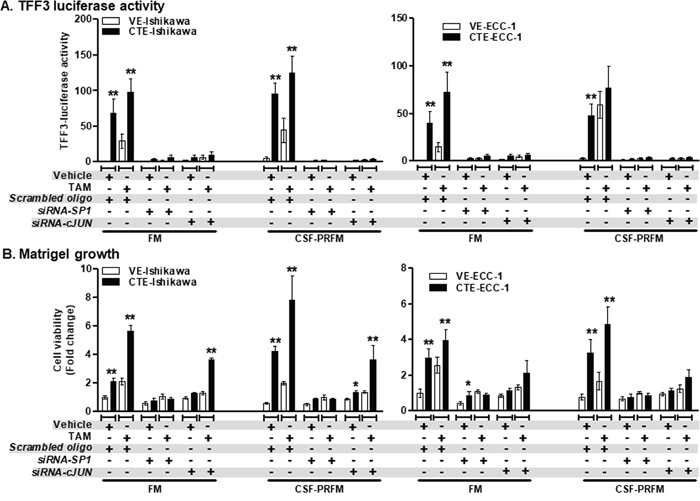
*siRNA*-mediated depletion of SP1 or *c-JUN* expression in ER+ EC cells abolished TAM-stimulated *promoter* activity of TFF3 and growth in Matrigel culture **(A)** *TFF3 promoter* activity and **(B)** growth in Matrigel of EC cells. EC cells (VE or CTE) were cultured in FM or CSF-PRFM. 5μM TAM was used to treat cells. Depletion of SP1 or c-JUN expression was achieved using transient-transfection of *si*-*RNA* directed to *SP1* or *c-JUN transcript* respectively, as described in materials and methods. Universal controls (scrambled oligo) were used for transfection control as described in materials and methods. The luciferase assay was performed as described in materials and methods. FM were 10%FBS, standard media conditions as per ATCC propagation instructions; and CSF-PRFM were charcoal striped 10% FBS, phenol-red free media. Statistical significance was assessed by using an unpaired two-tailed *Student's t test* (*P<0.05* was considered as significant) using GraphPad Prism5. Columns are mean of triplicate experiments; bars, ±*SD*. **P < 0.001, *P < 0.05.

To further confirm the involvement of SP1 and c-JUN transcription factors in TAM-stimulated TFF3 transcription and the resulting functional effects, *siRNA*-mediated depletion of SP1 or c-JUN was performed in the EC cells (Supplementary Figure 2D and 2E). CTE-Ishikawa cells exhibited increased TFF3 luciferase activities as compared to VE-Ishikawa cells when cultured in FM or CSF-PRFM (Figure 8A). On exposure to TAM, both CTE-Ishikawa and VE-Ishikawa cells exhibited increased TFF3 luciferase activities as compared to the respective vehicle exposed cells (Figure 8A). *siRNA*-mediated depletion of SP1 or c-JUN abrogated the higher TFF3 luciferase activities in CTE-Ishikawa cells and eliminated the TAM-stimulated increase in TFF3 luciferase activities in both CTE-Ishikawa and VE-Ishikawa cells (Figure 8A). Concordantly, CTE-Ishikawa cells exhibited increased 3D growth as compared to VE-Ishikawa cells when cultured in FM or CSF-PRFM (Figure 8B). On exposure to TAM, both CTE-Ishikawa and VE-Ishikawa cells exhibited increased 3D growth as compared to the respective vehicle exposed cells (Figure 8B). *siRNA*-mediated depletion of SP1 abrogated the higher 3D growth of CTE-Ishikawa cells and eliminated the TAM-stimulated increase in 3D growth of both CTE-Ishikawa and VE-Ishikawa cells (Figure 8B). However, *siRNA*-mediated depletion of c-JUN did not significantly decrease the higher 3D growth of CTE-Ishikawa cells nor eliminate the TAM-stimulated increase in 3D growth of CTE-Ishikawa cells (Figure 8B). In addition, the higher TFF3 luciferase activities in CTE-ECC-1 cells, and the tamoxifen-stimulated increase in TFF3-luciferase activities in both CTE-ECC-1 and VE-ECC-1 cells were abrogated by depletion of either SP1 or c-JUN (Figure 8A). This decrease in *TFF3 promoter* activities upon SP1 or c-JUN depletion was concordant with a corresponding abrogation of the enhanced 3D growth of the CTE-ECC-1 cells, and the TAM-stimulated 3D growth of both CTE-ECC-1 and VE-ECC-1 cells (Figure 8B).

In addition, we also examined for a potential association of TFF3, p-c-JUN, c-JUN, and SP1 protein expression in the cohort of EC patients used above in Figure 4B. IHC analysis showed that TFF3 expression positively correlated with expression of p-c-JUN, c-JUN, or SP1 in the EC cohort tumour specimen, as compared using Pearson *correlation* (*r*) summarized in Table 3.

**Table 3:** Correlation between expression of TFF3, p-c-JUN, c-JUN, and SP1 protein markers in EC cohort utilized in Figure 3B

Marker		TFF3	p-c-JUN	c-JUN	SP1
**TFF3**	*r*	1	0.427	0.303	0.383
	*p-value*	-	0.003	0.038	0.008
	*N*	-	47	47	47
**p-c-JUN**	*r*	0.427	1	-0.081	0.154
	*p-value*	0.003	-	0.588	0.302
	*N*	47	-	47	47
**c-JUN**	*r*	0.303	-0.081	1	0.281
	*p-value*	0.038	0.588	-	0.056
	*N*	47	47	-	47
**SP1**	*r*	0.083	0.154	0.281	1
	*p-value*	0.008	0.302	0.056	-
	*N*	47	47	47	-

Note: Cohort size (N)=47; *r*, *Pearson's chi-square* test was used to compare the differences between groups. Statistical significance was assessed by using an unpaired two-tailed *Student's t test* (*P < 0.05* was considered as significant) using GraphPad Prism 5.

### Inhibition of TFF3 or TFF3-mediated STAT3 signalling abrogates oncogenicity and invasion of ER+ EC cells owing to TAM-induction or forced expression of TFF3

**Figure 9:**
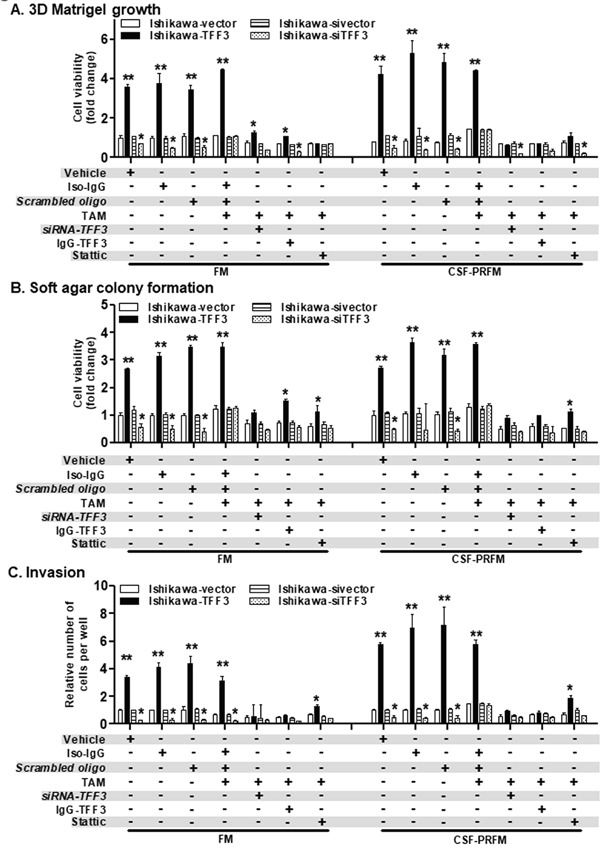
Inhibition of TFF3 or its downstream effector abrogates oncogenicity and invasion of Ishikawa cells owing to TAM-induction or forced expression of TFF3 **(A)** Growth in Matrigel culture. Ishikawa cells with either forced or depleted expression of TFF3 were seeded in 2% Matrigel and irregular colonies formed were counted after incubation for fourteen days, as described in materials and methods. Cells were cultured in FM or CSF-PRFM. Representative images of colonies formed in Matrigel were presented below. 5μM TAM and 2μM Stattic was used to treat cells. 10μg/ml each of IgG-TFF3 polyclonal antibody was used to treat cells. Cell viability was measured using Alamar Blue as described in materials and methods. **(B)** Soft agar colony formation. Ishikawa cells with either forced or depleted expression of TFF3 were seeded in 0.35% agarose and colonies (more than 50μM size) formed were counted after incubation for fourteen days, as described in materials and methods. Cells were cultured in FM or CSF-PRFM. Representative images of colonies formed in Matrigel were presented below. 5μM TAM and 2μM Stattic was used to treat cells. 10μg/ml each of IgG-TFF3 polyclonal antibody was used to treat cells. Cell viability was measured using Alamar Blue as described in materials and methods. **(C)** Trans-well cell invasion of Ishikawa cells with either forced or depleted expression of TFF3 as described in materials and methods. Cells were cultured in FM or CSF-PRFM. Representative images of colonies formed in Matrigel were presented below. 5μM TAM and 2μM Stattic was used to treat cells. 10μg/ml each of IgG-TFF3 polyclonal antibody was used to treat cells. FM were 10%FBS, standard media conditions as per ATCC propagation instructions; and CSF-PRFM were charcoal striped 10% FBS, phenol-red free media. Statistical significance was assessed by using an unpaired two-tailed *Student's t test* (*P<0.05* was considered as significant) using GraphPad Prism5. Columns are mean of triplicate experiments; bars, ±*SD*. **P < 0.001, *P < 0.05.

TFF3 has previously been reported to act through STAT3 to stimulate breast cancer metastasis [21]. To determine the role of TFF3-mediated STAT3 signalling in promoting the oncogenicity and invasion of ER+ EC cells, we performed cell functional assays using the STAT3 inhibitor, Stattic, in EC cells exposed to TAM (Figure 9 and Supplementary Figure 3). As previously observed, the forced expression of TFF3 increased; while the stable depletion of TFF3 decreased the 3D Matrigel growth, anchorage-independent growth, and invasion of the EC cells. Similarly, acute TAM treatment increased the 3D Matrigel growth, anchorage-independent growth, and invasion of the EC cells. The *siRNA*-mediated depletion or the monoclonal antibody-mediated inhibition of TFF3 in EC cells significantly abrogated the higher 3D Matrigel growth, anchorage-independent growth and invasion owing to TAM-induction or forced expression of TFF3 (Figure 9). Importantly, the inhibition of STAT3 by Stattic also reduced the enhanced 3D Matrigel growth, anchorage-independent growth and invasion of the EC cells owing to TAM-induction or forced expression of TFF3 (Figure 9). Similar directional changes was observed on 3D Matrigel growth, anchorage-independent growth and invasion of the ECC-1 cells after inhibition of TFF3 or STAT3 in the presence of acute TAM exposure (Supplementary Figure 3A, 3B and 3C). Therefore, TAM-stimulated oncogenicity and invasion of EC cells is dependent on TFF3-mediated STAT3 activation.

## DISCUSSION

TAM is a widely-used drug in the treatment of BC and long-term side effects include an increased risk for EC [9]. The increased risk of EC in TAM-treated BC patients ranges from 1.5 to 6.9–fold in different studies [13]. BC patients also tend to have an increased risk of endometrial proliferative disorders, thereby predisposing them to the development of EC upon TAM treatment [13]. Importantly, EC resulting from TAM treatment are often associated with poor clinical outcome and increased mortality [13]. A well-studied mechanism accounting for the agonistic effects of TAM in the endometrium is the preferential recruitment of co-activators by TAM-bound ERα, being attributed to differential expression of ERα co-factors in different tissues [13]. However, not all ERα target genes are upregulated in the endometrium upon TAM treatment, suggesting that this mechanism only partially accounts for the agonistic effect of TAM in the endometrium. The current understanding of TAM-induced EC remains limited, hence this reinforces the need to identify novel strategies to treat EC and EC that arises from the use of TAM [13]. Chronic exposure to TAM has been previously linked to increases in cell survival, anchorage independent growth, and invasiveness of oestrogen-responsive EC cells [13]. Clinical transcriptomics in EC reveal several genes that are associated with TAM treatment and could be used to predict survival rates post-TAM treatment [35]. Herein, we reported that both acute and chronic TAM treatment resulted in increased expression of the ER-responsive TFF3 gene, which in turn promoted EC oncogenicity.

An *in silico* promoter analysis performed by Borthwick *et al.* (2003) has identified two candidate *ERE sequences* in the first -1kb of the *TFF3 promoter* [22]. A study by Theodorou *et al.* (2013) has shown that GATA1 modulates enhancer accessibility and ERα binding that regulates the *transcription* of ERα-regulated genes including TFF3 [36]. Similarly, it has been reported that TFF3 expression is regulated by ERα binding to ER binding sites (ERBSs) brought into vicinity of the *TFF3 locus* [37]. Given that the effect of chronic TAM exposure on TFF3 expression is maintained in the absence of TAM, and the literature evidence for epigenetic regulation of TFF3 expression at the *core promoter* (-700bp) level [28, 38, 39], we chose to examine the *proximal promoter* for the ability of TAM to epigenetically regulate TFF3 expression. In this study, we have reported an epigenetic mechanism of TAM-stimulated upregulation of TFF3 through hypomethylation of its *proximal promoter* region. In general, methylation of CpG sites in gene promoters can lead to downregulation or silencing of gene expression while demethylation results in the opposite. DNA hypomethylation is a ubiquitous phenomenon associated with various types of cancers [38]. Hypomethylation of the *TFF3 promoter* region, resulting in increased TFF3 expression, has been observed in hepatocellular carcinoma [28], and prostate cancer [39]. In contrast, retinoblastoma cell lines, with low levels of TFF3 expression, exhibited hypermethylation of the *TFF3 promoter* in a DNA methyltransferase 1 dependent manner [40]. Hence, a strong association between hypomethylation of the *TFF3 promoter* with high expression of TFF3 has been previously established [41]. Herein, both chronic and acute exposure of EC cells to TAM resulted in decreased methylation of the *TFF3 promoter* region corresponding to binding sites for the transcription factors c-JUN and SP1. We showed that the TAM-stimulated expression of TFF3 was at least partially c-JUN and/or SP1 dependent in EC cells. Concordantly, TFF3 expression in EC tumour samples positively correlated with the levels of SP1, and the activity and levels of phospho c-JUN. c-JUN has been previously implicated in the progression of BC showing an increase in invasive potential of the cancer cells that is associated with angiogenesis and proliferation [42]. SP1 is a transcription factor containing a zinc-finger that recognises GC-rich regions and regulates many genes in the cell, including proto-oncogenes and tumour suppressor genes [43]. Amongst its many targets, SP1 can also work together with ERα to regulate the expression of many E2-responsive genes, as reported in a study utilising BC cells [44]. A previous study of the human *TFF3 promoter* in colon cancer cells had also found that the transcription of *TFF3* was precisely controlled by an SP1 binding site, corroborating the results obtained in our experiments [45]. Hence, there appears to be a combinatorial control of TFF3 expression by chronic tamoxifen exposure in EC cells; the hypomethylation of the *TFF3* promoter to potentially increase availability of TF binding sites as well as the specific increase in phospho-c-JUN activity and SP1 expression that interact with the binding sites on *TFF3* promoter and stimulate transcription of *TFF3.* TFF3 overexpression also leads to enhanced phospho c-JUN activates and SP1 expression at the *TFF3* promoter site, in turn leading to upregulation of TFF3 expression. This may suggest that TFF3 can transcriptionally upregulate its own expression in a positive feedback loop through increasing the activity and levels of its transcription factors, namely SP1 and/or c-JUN. A similar observation of TFF3 positively regulating its own expression has been previously reported, in which TFF3, being an oestrogen-responsive gene, was found to increase ERα transcriptional activity [17]. Furthermore, the possible TFF3-mediated upregulation and/or activation of SP1 and c-JUN transcription factors further supports this notion as TFF3 has been suggested to be a promiscuous ligand which activates multitude of signalling pathways including EGFR, HER2, MET, CXCR4/7, and RTKs [21, 34, 46, 47]. This mode of TFF3 transcriptional regulation through a positive feedback loop is an interesting finding that warrants further investigation.

In this study, we demonstrated that the elevated TFF3 expression upon acute or chronic TAM exposure is responsible for increased growth and invasion of the EC cells. Consistently, forced expression of TFF3 stimulated cell proliferation and survival, 3D cell growth, anchorage-independent growth, and invasive and metastatic potential of EC cells. These observations have been complemented by TFF3 depletion studies showing opposite trends. In addition to EC, TFF3 has previously been shown to enhance cell proliferation, survival, oncogenicity, invasion and metastasis of BC [17, 21], prostate cancer [29], cervical cancer [23] and hepatocellular carcinoma [48]. Consistent with TFF3-stimulated oncogenicity and EMT, we observed that TFF3 promotes the expression of cell cycle progression, anti-apoptotic, mesenchymal and metastasis-associated genes, and suppresses the expression of cell-cycle inhibitors, pro-apoptotic and epithelial genes in EC cells. A similar trend of TFF3-regulated gene expression has previously been reported in breast, prostate, cervical and hepatocellular cancers [17, 21, 23, 29, 48]. In addition, we herein observed a graded increase in TFF3 expression from normal endometrium to endometrial hyperplasia to EC. A significantly higher percentage of EC patients exhibited high TFF3 expression as compared to low TFF3 expression. This is consistent with a previous study, which reported increased TFF3 gene and protein expression, and serum TFF3 protein levels in high grade endometroid EC as compared to normal endometrium [24]. The overexpression of TFF3 has also been reported clinically in breast [25], prostate [16, 39, 49, 50], lung [30, 51], gastric [52–54] and liver [28, 55, 56] cancers. Furthermore, in our cohort of EC patients, TFF3 expression is positively associated with myometrial invasion, indicating that TFF3 likely promotes EMT leading to cancer progression. We have also previously reported a positive correlation of TFF3 expression with tumour size, lymph node metastasis, higher stage and poorer survival outcome in ER+ BC [21].

Herein, we also demonstrated that the TFF3-stimulated oncogenicity of EC cells is at least partially dependent on STAT3 activity. TFF3-mediated activation of STAT3 has also been previously reported to stimulate progression of different cancers [21, 25]. Our group has earlier shown that TFF3 increased STAT3 activity, which resulted in downregulation of E-cadherin, stimulated invasion and metastasis of ER+ mammary carcinomas [21]. This is supported by a recent study in which TFF3 decreased E-cadherin expression in a manner dependent on STAT3 activity, hence promoting the invasiveness of cervical cancer cells [23]. Similarly, it has been demonstrated that the TFF3- and VEGF-stimulated invasion and growth of colorectal cancer cells is dependent on STAT3 activation [57]. In addition, our previous study has shown that TFF3 promotes STAT3-dependent transcription of IL8, thereby stimulating angiogenesis in mammary carcinoma through the IL-8/CXCR2 axis [27].

In summary, TFF3 is a potential therapeutic target in EC and in TAM-associated EC. Importantly, TFF3 also promotes BC progression, decreases anti-oestrogen sensitivity and mediates anti-oestrogen resistance. The present study provides a compelling rational to explore therapeutic agents to inhibit TFF3 in EC and combination regimens of TAM with TFF3-inhibiting agents for hormone dependent BC, thereby preventing TAM-stimulating effects in endometrial tissues.

## MATERIALS AND METHODS

### Cell culture and reagents

The human EC cell lines, Ishikawa, ECC-1, RL95-2 and AN3, were obtained from the American Type Culture Collection (ATCC, Rockville, MD) and were cultured as per ATCC propagation instructions. Stable cell lines were freshly generated as previously described [21, 32], briefly, the human EC cell lines were stably transfected with either *pIRESneo3* containing the *TFF3 cDNA* (designated as Ishikawa-TFF3 and ECC-1-TFF3) or a *pSilencer* containing a *siRNA* targeting *TFF3* (designated as Ishikawa-siTFF3 and ECC-1-siTFF3), using FuGENE 6 (Promega, Singapore). Control cells lines were transfected with the empty *pIRESneo3* vector (designated Ishikawa-vector and ECC-1-vector) or the negative control *siRNA* plasmid (Ambion) as *siRNA* control (designated Ishikawa-sivector and ECC-1-sivector). To generate an *in vitro* model of chronic TAM stimulation, Ishikawa and ECC-1 cells were first exposed to 0.5μM of TAM for six weeks and then to 1μM of TAM for twelve weeks. Ishikawa and ECC-1 cells were cultured in DMEM (Hyclone, USA) supplemented with 10% heat inactivated fetal bovine serum (FBS) (Hyclone, USA), 100 IU/mL penicillin and 100μg/mL streptomycin (Invitrogen, USA) as per ATCC propagation instructions. These cells were designated as chronic TAM exposure (CTE)-Ishikawa and CTE-ECC-1 cells. Concomitantly, Ishikawa and ECC-1 cell line were also exposed to DMSO (solvent) for similar periods and were cultured as per ATCC propagation instructions. These EC cells were designated as vehicle exposure (VE)-Ishikawa and VE-ECC-1 cells. To generate oestrogen-depleted experimental condition, the cells were cultured in phenol red-free media supplemented with charcoal-stripped 10%FBS (CSF-PRFM). Prior to TAM or vehicle exposure, EC cells were cultured in CSF-PRFM for twelve hours. Tamoxifen, Doxorubicin (Dox) and STAT3 activity inhibitor, Stattic, were purchased from Sigma-Aldrich (Singapore).

### Patient tissue microarrays and IHC

The patient cohort used herein consists of 47 EC, 20 SEH, 12 CEH and 38 specimens of normal endometrium that underwent surgery at the First Affiliated Hospital of Anhui Medical University (AMU) (Hefei, Anhui, People's Republic of China) between 2001 and 2002 as previously described [58]. The Institutional Review Board of AMU approved the protocol for the use of patient specimens in this study [58]. Patient consent forms were obtained from all patients in accordance with the Declaration of Helsinki [58]. IHC analysis was performed as previously described [58] using rabbit anti-TFF3 was obtained from Abcam, Cambridge, MA; rabbit anti-SP1, rabbit anti-p-c-JUN and rabbit anti-c-JUN antibodies wereobtained from Cell Signaling Technology, Singapore [17]. The details of the cohort and IHC scoring methodology have previously been described in detail [30, 58, 59].

### Plasmids and luciferase assay

Human *TFF3* expression and *siRNA plasmid constructs* have been previously described [17]. The *TFF3*, *SP1* and *c-JUN Stealth RNAi duplexes* and *Stealth RNAi* negative control *duplexes* (Invitrogen, CA) were transfected into EC cells by FuGene X-tremeGENE 9 (Roche Applied Science, Singapore) according to the manufacturer's instructions. The *ERE luciferase reporter* have been previously described [17]. The 5′-flanking region of *human TFF3* (Genbank Accession NM_003226.3) was amplified by *PCR* from a sample of *genomic DNA* from Ishikawa cells using the primers, 5′- GCTAGCCGTGCTTAGATCCAGAGAG-3′ and 5′- CTCGAGAGGACCAGCCCCAGCAT-3′, which are engineered to contain a *Nhel* and an *Xhol* site respectively. The PCR product was gel-purified and cloned into the pGEM-T Easy vector (Promega, Madison, WI, USA), using the T-A cloning strategy. The *TFF3 promoter* (-700 to +50 bp) fragments were then cloned into the *pGL3-basic* luciferase reporter vector (Promega, USA). Site-directed mutagenesis was performed using the *Q5 DNA site-directed* mutagenesis kit (NEB, USA) following the manufacturer's instructions. We generated four single site mutant *TFF3 luciferase reporter constructs* based on the parental *TFF3 luciferase reporter construct* using *pGL3-basic* as backbone vector. The primers for mutating the four transcription factor binding sites (FOXA2, SOX5, c-JUN and SP1) were shown in Supplementary Figure 4. Following the *PCR* process, the amplified product was added directly to a unique Kinase-Ligase-DpnI (KLD) enzyme mix for rapid circularization and removal of the original template at room temperature. Mutations were verified through single-pass *DNA* sequencing.

Luciferase assays were performed as previously described [60]. Briefly, transfections were carried out in triplicate using 1μg of the appropriate luciferase reporter construct or empty vector along with 0.1μg of *Renilla* luciferase construct as control for transfection efficiency. Luciferase activities were assayed 24 hour after transfection using the Dual Luciferase Assay System (Promega Corp, Madison, WI).

### Bisulfite modification

*Genomic DNA* was prepared from EC cells by the standard method of proteinase K digestion and phenol/chloroform/isoamyl alcohol extraction. *Genomic DNA* was bisulfite-treated using CpGenome™ Turbo Bisulphite Modification kit from Millipore, CA. with some modification. Briefly, 10μg of *genomic DNA* from each samples of EC cells were digested with EcoRV and then denatured. Freshly prepared sodium bisulfite (pH5.0, 1020μl, 3.6M) and hydroquinone (60μl, 10mM) was added with a final volume of 1200μl and then the DNA solution incubated for 16h at 55°C. DNA was purified using the DNA Gel Extraction System (Qiagen, Singapore). After denaturation by NaOH and neutralization, the *DNA* was precipitated and resuspended in 50μl of water (DNAse free). PCR primers that amplify both *methylated DNA* and *unmethylated DNA* (Supplementary Figure 5) were designed using the Methprimer software (http://www.urogene.org/methprimer/). PCR conditions were summarized in Supplementary Figure 5. PCR products were assessed using electrophoresis with a 2% agarose gel and visualized.

### ChIP assay

*ChIP* assay was performed as previously described [61]. Briefly, EC cells were cultured in FM or CSF-PRFM and treated with 5μM TAM or vehicle (DMSO). Cells were then fixed, harvested and sonicated. 5μg of SP1 monoclonal antibody (ChIP-grade), c-JUN monoclonal antibody (ChIP-grade) or Rabbit IgG from Cell Signaling Technology, Singapore, was used in this assay. The precipitated *DNA* was purified and assessed by *quantitative-PCR* (*qPCR*) using c-JUN-FP: 5’GGAAAGACAAGGGAATCTCTGTGT3’, c-JUN-RP: 5’AGGCTGCTTCATCCCAGATC 3’; SP1-FP: 5’TGGGGCTCTCTTGTCATGG 3’ SP1-RP: 5’CCTGGTGTAGTGTGCAGGAG 3’.

### PCR and qPCR

Total *RNA* was isolated from cells (cultured in 10% fetal bovine serum) using TRIzol Plus RNA Purification system as previously described [32]. DNase I treatment, reverse transcription, *PCR*, and *qPCR* assays were performed as previously described [32]. Gene expression analysis was performed as previously described [32].

### Immunoblot

Immunoblot analysis and IHC was performed as previously described [32], using rabbit anti-TFF3, rabbit anti-pSTAT3, and mouse anti-STAT3 was obtained from Abcam, Cambridge, MA. While the mouse anti-CCND1, mouse anti-BCL2, mouse anti-β-ACTIN antibodies were obtained from Santa Cruz Biotechnology, CA.

### Oncogenicity assays

Cell migration and invasion assays were performed using BD BioCoat Matrigel invasion chambers (BD Biosciences, Bedford, MA) according to the manufacturer's instructions and as previously described [32]. The colony scattering assay was performed as previously described [62]. ApoTox-Glo^™^ Triplex Assay Kit from Promega was performed according to the macufacturer's instructions and as previously described [63]. Cell functional assays, including an AlamarBlue® viability assay, anchorage-independent growth (soft agar colony formation and foci formation), 2D and 3D morphogenesis, and wound-healing assay were performed as previously described [32]. The collagen I adhesion assay was performed on Collagen I substrate coated plate following manufacturer's instructions (Invitrogen, Singapore). The endothelial cell adhesion and endothelial trans-migration assays were performed as previously described [62].

### Xenograft assay

The *in vivo* work was carried out following the animal care protocol USTCACUC1301013 which was approved by The Institutional Animal Care and Ethics Committee of The University of Science and Technology of China. Xenograft assays were performed as previously described. Briefly, Ishikawa-vector or Ishikawa-TFF3 cells (1.5×10^8^) were suspended in 100μl PBS and injected subcutaneously (sc) into 3- to 4-wk-old BALBc nu/nu mice (Shanghai Slaccas Co., Shanghai, China). The latency of the tumours in the nude mice in this study was around 1 week, and tumours were harvested 40 days after inoculation. Tissue samples of the primary tumours were fixed in 4% paraformaldehyde and stained with haematoxylin and eosin for histological assessment. For BrdU analysis, mice were injected intraperitoneally (ip) with BrdU (100 mg/kg) 6 h before euthanasia. Nuclear BrdU was detected in sections using an UltraSensitive S-P Kit for BrdU detection (FuZhou MAIXIN BIO Co., FuZhou, China) according to the manufacturer's instructions, and slides were counterstained with Gill's haematoxylin. For TUNEL analysis, paraffin-embedded tumor tissue sections were deparaffinized and rehydrated, followed by treatment with 15 mg/liter proteinase K for 30 min. Apoptotic nuclei in the section were analyzed with an *in situ* Cell Death Detection Kit, POD (Roche, Indianapolis, IN) according to the manufacture's guide. The number of TUNEL-positive cells in tumor sections was determined in at least three independent high-resolution fields selected randomly, and around 800 cells were counted per slide. IHC was performed as described in the previous section.

### Statistics

Statistical analyses of patient's samples was performed using SPSS software (version 13.0; SPSS, Chicago, IL), as previously described [58]. Briefly, differences between groups were compared using *Pearson's chi-square* test for qualitative variables and *Student's t-test* for continuous variables. All numerical data are expressed as mean ±SD from a representative experiment performed in triplicate. Statistical significance was assessed by using an unpaired two-tailed *Student's t* test (P < 0.05 was considered as significant) by GraphPad Prism 5 (GraphPad Software, Inc, La Jolla, CA).

## SUPPLEMENTARY MATERIALS FIGURES


