# Supplementary material for: Hypomethylation associated enhanced transcription of trefoil factor-3 mediates tamoxifen-stimulated oncogenicity of ER+ endometrial carcinoma cells
Source: Oncotarget. 2017 Aug 24;8(44):77268–91. doi: 10.18632/oncotarget.20461 (PMC5652779; doi:10.18632/oncotarget.20461)
Supplement: Supplementary file 1 [file oncotarget-08-77268-s001.pdf]

Hypomethylation associated enhanced transcription of trefoil factor-3 mediates tamoxifen-stimulated oncogenicity of ER+ endometrial carcinoma cells

SUPPLEMENTARY MATERIALS

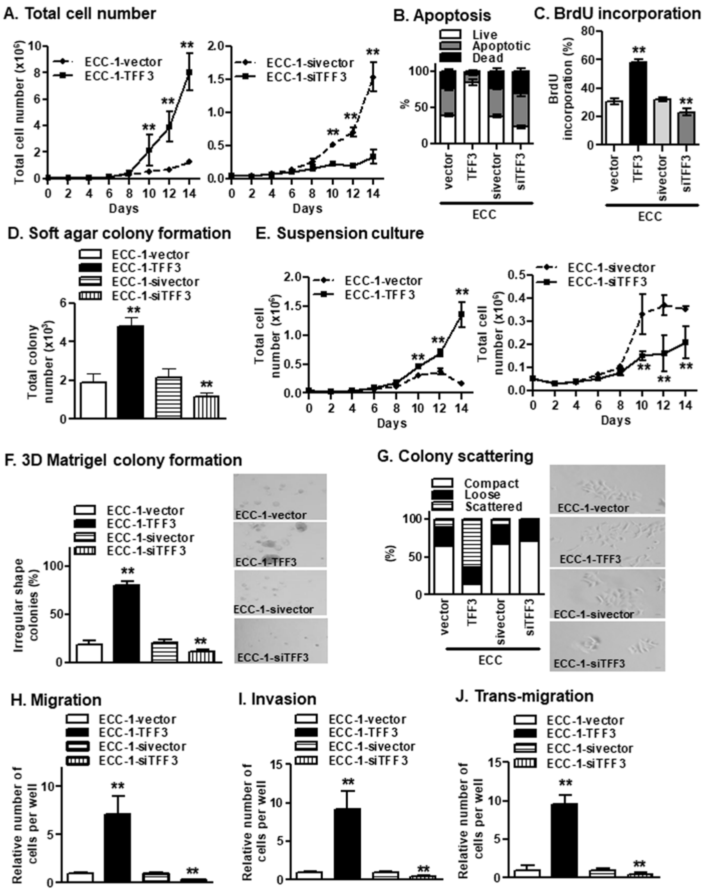

Supplementary Figure 1: Forced expression of TFF3 in ECC-1 cells stimulates oncogenicity. **(A)** Total cell number assay. ECC-1 cells with either forced or depleted expression of TFF3 were cultured in 10%FBS, standard media conditions as per ATCC propagation instructions, as described in materials and methods. **(B)** Apoptosis. ECC-1 cells with either forced or depleted expression of TFF3 were fixed and were stained for the assessment of early and late phase of apoptotic cell death using fluorescein isothiocyanate-conjugated annexin V (AV) and propidium iodide (PI), as described in materials and methods. **(C)** S-phase entry. The percentage of bromodeoxyuridine (BrdU)-positive cell nuclei relative to the total number of cell nuclei in ECC-1 cell with either forced or depleted expression of TFF3 was determined, as described in materials and methods. **(D)** Soft agar colony formation. ECC-1 cells were seeded in 0.35% agarose and colonies (more than 50µm size) formed were counted after incubation for fourteen days, as described in materials and methods. **(E)** Growth in suspension culture. ECC-1 cells were seeded in low adherent plate and colonies (more than 50µm size) formed were counted after incubation for fourteen days, as described in materials and methods. **(F)** Growth in Matrigel culture. ECC-1 cells were seeded in 2% Matrigel and irregular colonies formed were counted after incubation for fourteen days, as described in materials and methods. Representative images of colonies formed in Matrigel were presented below. **(G)** Colony scattering assay. Distribution of compact, loose, and scattered colonies of ECC-1 cells with either forced or depleted expression of TFF3. Cells were plated at colony-forming conditions. One hundred colonies in each sample were categorized after scoring phase contrast images into three categories: compact (in which >90% of cells in a colony have cell-cell junctions), loose (in which 50–90% of cells form junctions), and scattered (in which <50% of cells form junctions), as described in materials and methods. Representative images of colonies formed were presented below. **(H)** Migration. Capacity of ECC-1 cells with either forced or depleted expression of TFF3 to migrate through membrane in trans-well chamber, as described in materials and methods. **(I)** Invasion. Capacity of ECC-1 cells with either forced or depleted expression of TFF3 to invade and migrate through Matrigel coated membrane in trans-well chamber, as described in materials and methods. **(J)** Trans-migration. Capacity of ECC-1 cells with either forced or depleted expression of TFF3 to transmigrate through a HMEC-1 layer, as described in materials and methods. Statistical significance was assessed by using an unpaired two-tailed *Student's t test* (*P<0.05* was considered as significant) using GraphPad Prism5. Columns or points are mean of triplicate experiments; bars, ±*SD*. $^{**}P < 0.001$, $^{*}P < 0.05$.

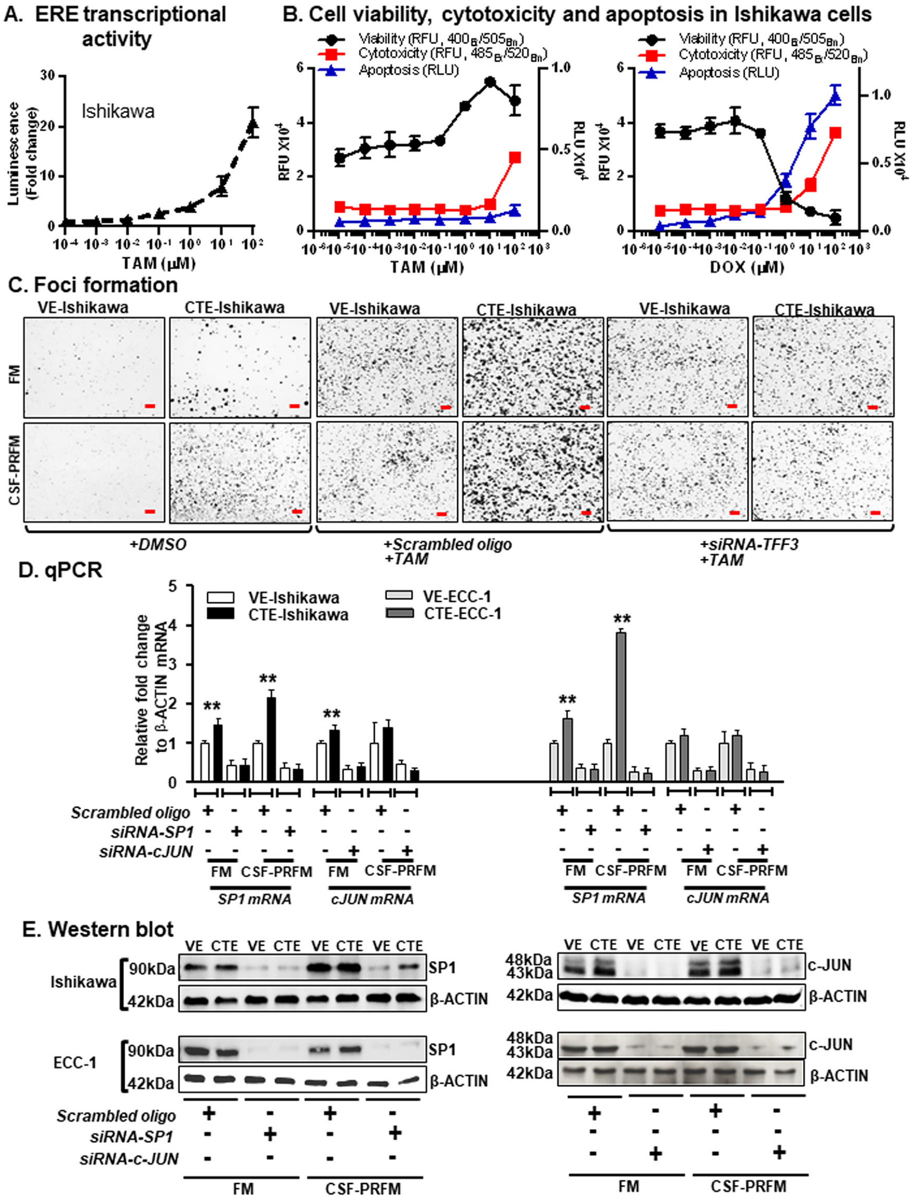

Supplementary Figure 2: (A) *Oestrogen response element (ERE) transcriptional* activity was measured of Ishikawa cells after exposure to increased doses of TAM. Universal controls (scrambled oligo) were used for transfection control as described in materials and methods. The luciferase assay was performed as described in materials and methods. **(B)** Cell viability, cytotoxicity, or caspase 3/7 activities was assessed in Ishikawa cells using ApoTox-Glo™ Triplex Assay Kit from Promega, as described in materials and methods. Cell viability fluorescence is measured at $400_{Ex}/505_{Em}$, cytotoxicity fluorescence is measured at $485_{Ex}/520_{Em}$, while apoptosis (caspase 3/7 activities) is determined by luminescence measurement. RFU, relative fluorescence unit; RLU, relative luminescence unit; TAM, Tamoxifen; Dox, Doxorubicin (positive control) **(C)** TAM-stimulated foci formation abolished in Ishikawa cells after *siRNA*-mediated depletion of TFF3. Foci formation were performed as described in materials and methods. **(D)** *qPCR* analysis was used to evaluate the *mRNA* levels of *c-JUN* and *SP1* genes in EC cells. EC cells (VE or CTE) were cultured in FM (10%FBS, standard media conditions as per ATCC propagation instructions) or CSF-PRFM (charcoal striped 10% FBS, phenol-red free) media. Depletion of *c-JUN* or *SP1* expression was achieved using transient-transfection of *siRNA* directed to *c-JUN* or *SP1 transcript* as described in materials and methods. *qPCR* analysis were performed as described in materials and methods. **(E)** Western blot analysis was used to assess the levels of c-JUN and SP1 in EC cells with VE or CTE. EC cells (VE or CTE) were cultured in FM (10%FBS, standard media conditions as per ATCC propagation instructions) or CSF-PRFM (charcoal striped 10% FBS, phenol-red free) media. 5μM TAM was used to treat cells. Depletion of *c-JUN* or *SP1* expression was achieved using transient-transfection of *siRNA* directed to *c-JUN* or *SP1 transcript* as described in materials and methods. Soluble whole cell extracts were run on a SDS-PAGE and immunoblotted as described in materials and methods. β-ACTIN was used as input control for cell lysate. The predictable sizes of detected protein bands in kDa are shown on the *left side.*

Statistical significance was assessed by using an unpaired two-tailed *Student's t test* (*P<0.05* was considered as significant) using GraphPad Prism5. Columns are mean of triplicate experiments; bars, ±*SD*. $^{**}P < 0.001$, $^{*}P < 0.05$.

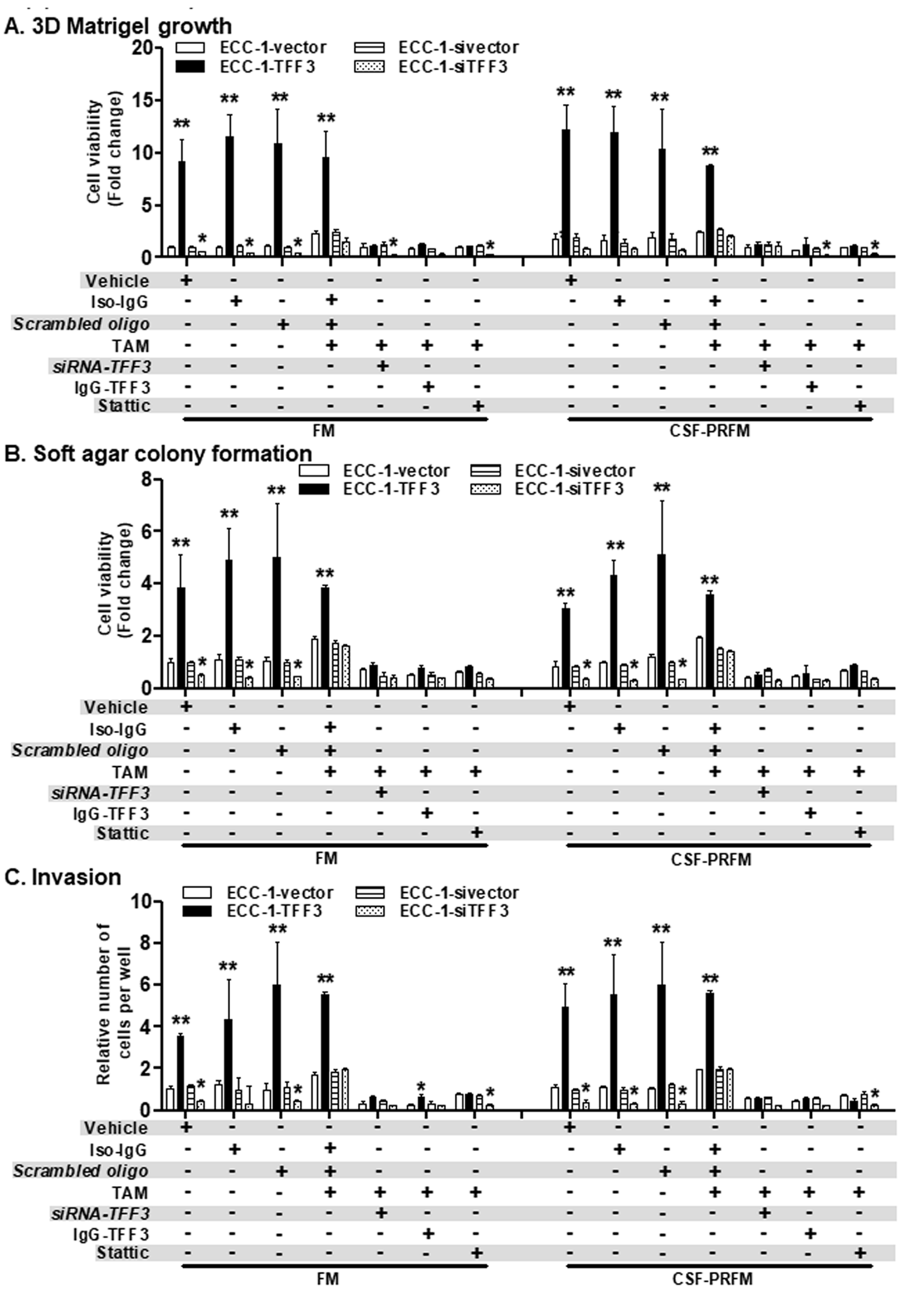

Supplementary Figure 3: Inhibition of TFF3 or its downstream effector abrogates oncogenicity and invasion of ECC-1 cells owing to TAM-induction or forced expression of TFF3. **(A)** Growth in Matrigel culture. ECC-1 cells with either forced or depleted expression of TFF3 were seeded in 2% Matrigel and irregular colonies formed were counted after incubation for fourteen days, as described in materials and methods. Cells were cultured in FM or CSF-PRFM. Representative images of colonies formed in Matrigel were presented below. 5µM TAM and 2µM Stattic was used to treat cells. 10µg/ml each of IgG-TFF3 polyclonal antibody was used to treat cells. Cell viability was measured using Alamar Blue as described in materials and methods. **(B)** Soft agar colony formation. ECC-1 cells with either forced or depleted expression of TFF3 were seeded in 0.35% agarose and colonies (more than 50µm size) formed were counted after incubation for fourteen days, as described in materials and methods. Cells were cultured in FM and CSF-PRFM. Representative images of colonies formed in Matrigel were presented below. 5µM TAM and 2µM Stattic was used to treat cells. 10µg/ml each of IgG-TFF3 polyclonal antibody was used to treat cells. Cell viability was measured using Alamar Blue as described in materials and methods. **(C)** Trans-well cell invasion of ECC-1 cells with either forced or depleted expression of TFF3 as described in materials and methods. Cells were cultured in FM or CSF-PRFM. Representative images of colonies formed in Matrigel were presented below. 5µM TAM and 2µM Stattic was used to treat cells. 10µg/ml each of IgG-TFF3 polyclonal antibody was used to treat cells.
FM were 10%FBS, standard media conditions as per ATCC propagation instructions; and CSF-PRFM were charcoal striped 10% FBS, phenol-red free media. Statistical significance was assessed by using an unpaired two-tailed *Student's t test* (*P<0.05* was considered as significant) using GraphPad Prism5. Columns are mean of triplicate experiments; bars, ±*SD*. ***P < 0.001*, **P < 0.05*.

TF binding site	Primer sequence
FOXA2	Forward Primer: 5’ GTGTACCATGTTTTTACTAAC 3’<br>Reserved Primer: 5’ CAAAAGAGCCTTTCCTATC 3’
SOX5	Forward Primer: 5’ AGAGCAGCTGTGCAAACAAC 3’<br>Reserved Primer: 5’ CTTGGGGAAGGCTCTCCC 3’
c-JUN	Forward Primer: 5’ CTCAGAGCTGCCTGTCTC 3’<br>Reserved Primer: 5’ CAGAGATTCCTTGTCTTTCC 3’
SP1	Forward Primer: 5’ TGGTCCTGCCACCCCACA 3’<br>Reserved Primer: 5’ TCCGTTCCATCTCAGCCTCC 3’

Supplementary Figure 4: Primer sequence of transcription factor (TF) binding site.

Gene		Primer sequence	PCR condition<br>96^{0}C for 10min<br>96^{0}C for 30sec<br>Annealing temperature "X" for 30sec<br>73^{0}C for 30sec.	PCR product (bp)
**251M**	Forward	AGAGTTGTTTGTTTTCGAGGTCGAT	**Annealing temperature: 70^{0}C**	**231**
	Reverse	ACGAATTCCAATTCTAATTCCGAAT		
**251UM**	Forward	AGAGTTGTTTGTTTTTGAGGTTGAT	**Annealing temperature: 70^{0}C**	**231**
	Reverse	ACAAATTCCAATTCTAATTCCAAAT		
**50M**	Forward	AAATAATTTAGAGTAGTTGTGTAAATAACG	**Annealing temperature: 65^{0}C**	**100**
	Reverse	CATAACCACCGTAAACTCCGA		
**50UM**	Forward	AATAATTTAGAGTAGTTGTGTAAATAATG	**Annealing temperature: 65^{0}C**	**102**
	Reverse	ACCATAACCACCATAAACTCCAA		

Note: Methylated, M; Unmethylated, UM; base pair, bp; Annealing temperature, X.

Supplementary Figure 5: Methylated/Unmethylated primer sequences and PCR condition.
